# Characterization of human anti-EpCAM antibodies for developing an antibody–drug conjugate

**DOI:** 10.1038/s41598-023-31263-x

**Published:** 2023-03-14

**Authors:** Hiroyuki Satofuka, Yayan Wang, Kyotaro Yamazaki, Shusei Hamamichi, Takeshi Fukuhara, Abdur Rafique, Nana Osako, Iori Kanazawa, Takeshi Endo, Naomi Miyake, Kazuhisa Honma, Yuichi Nagashima, Genki Hichiwa, Kazuto Shimoya, Satoshi Abe, Takashi Moriwaki, Yasufumi Murakami, Xu Gao, Hiroyuki Kugoh, Mitsuo Oshimura, Yuji Ito, Yasuhiro Kazuki

**Affiliations:** 1 Chromosome Engineering Research Center, Tottori University, 86 Nishi-cho, Yonago, Tottori 683-8503 Japan; 2 Department of Chromosome Biomedical Engineering, School of Life Science, Faculty of Medicine, Tottori University, 86 Nishi-cho, Yonago, Tottori 683-8503 Japan; 3 Department of Biochemistry and Molecular Biology, Harbin Medical University, Harbin, 150081 Heilongjiang China; 4 Biomedical Science, Institute of Regenerative Medicine and Biofunction, Graduate School of Medical Science, Tottori University, 86 Nishi-cho, Yonago, Tottori 683-8503 Japan; 5 Division of Genome and Cellular Functions, Integrated Medical Sciences, Graduate School of Medical Science, Tottori University, 86 Nishi-cho, Yonago, Tottori 683-8503 Japan; 6 Research Institute for Diseases of Old Age, Juntendo University School of Medicine, 2-1-1 Hongo, Bunkyo-ku, Tokyo, 113-8421 Japan; 7 Department of Neurology, Juntendo University Graduate School of Medicine, 2-1-1 Hongo, Bunkyo-ku, Tokyo, 113-8421 Japan; 8 Department of Chemistry and Bioscience, Graduate School of Science and Engineering, University of Kagoshima, 1-21-40 Korimoto, Kagoshima, 890-0065 Japan; 9 Trans Chromosomics Inc., 86 Nishi-cho, Yonago, Tottori 683-8503 Japan; 10 Order-Made Medical Research Inc., 5-4-19 Kashiwanoha, Kashiwa, Chiba 277-0882 Japan

**Keywords:** Biotechnology, Biologics, Antibody therapy

## Abstract

We previously generated fully human antibody-producing TC-mAb mice for obtaining potential therapeutic monoclonal antibodies (mAbs). In this study, we investigated 377 clones of fully human mAbs against a tumor antigen, epithelial cell adhesion molecule (EpCAM), to determine their antigen binding properties. We revealed that a wide variety of mAbs against EpCAM can be obtained from TC-mAb mice by the combination of epitope mapping analysis of mAbs to EpCAM and native conformational recognition analysis. Analysis of 72 mAbs reacting with the native form of EpCAM indicated that the EpCL region (amino acids 24–80) is more antigenic than the EpRE region (81–265), consistent with numerous previous studies. To evaluate the potential of mAbs against antibody–drug conjugates, mAbs were directly labeled with DM1, a maytansine derivative, using an affinity peptide-based chemical conjugation (CCAP) method. The cytotoxicity of the conjugates against a human colon cancer cell line could be clearly detected with high-affinity as well as low-affinity mAbs by the CCAP method, suggesting the advantage of this method. Thus, this study demonstrated that TC-mAb mice can provide a wide variety of antibodies and revealed an effective way of identifying candidates for fully human ADC therapeutics.

## Introduction

Transchromosomic mice carrying mini-chromosomes with human immunoglobulin (Ig) loci have contributed to the generation and isolation of fully human therapeutic monoclonal antibodies (mAbs)^1,2^. Recently, we reported that human antibody-producing transchromosomic (TC-mAb) mice, which stably maintained a mouse-derived engineered chromosome (mouse artificial chromosome: MAC)^3^ containing the entire human Ig heavy and kappa light chain loci (*IGH* and *IGK*) (designated IGHK-NAC) in a mouse Ig knockout background^4^, produced more subsets of antigen-specific plasmablasts and plasma cells than wild-type mice, leading to highly efficient hybridoma production. Although MAC-based, human antibody-producing transchromosomic mice are expected to be useful, the distribution of epitopes and functions among the obtained mAbs has not been validated in detail.

Epithelial cell adhesion molecule (EpCAM)—a homophilic cell–cell adhesion glycoprotein—is a well-known tumor antigen expressed on epithelial tumors, circulating tumor cells, and cancer stem cells^5,6^. Therefore, diagnostic and therapeutic anti-EpCAM antibodies have been generated, including those that are approved for detecting circulating tumor cells^7–9^, and further developed into therapeutic agents^8^ against solid tumors, including colon and breast cancers^10,11^.

**Figure 1:**
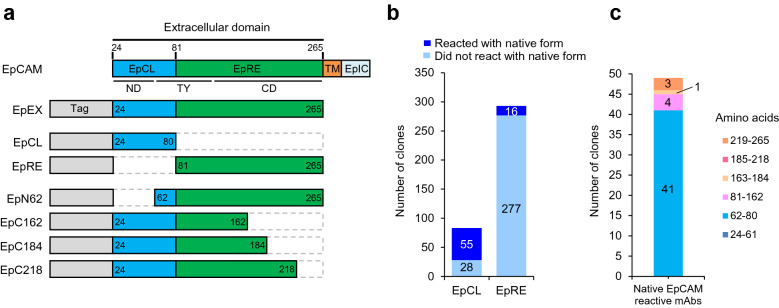
Production of anti-epithelial adhesion molecule (EpCAM) monoclonal antibodies (mAbs) in transchromosomic (TC-mAb) mice. (**a**) Schematics of partially fragmented EpCAM proteins. The EpCL region, illustrated in blue, contains the N-terminal domain (ND) and a part of the thyroglobulin type-1 domain (TY), whereas the EpRE region, illustrated in green, contains parts of TY and the cysteine-poor region (CD). All EpCAM protein fragments used in this study lack TM and EpIC regions. Truncated fragments are shown as dotted gray lines. The numbers in the box indicate the amino acid number to be translated at the start or end of the truncated protein. (**b**) Quantitative comparison of the mAbs and their binding regions. The numbers of mAbs that were reactive with the EpCAM native form (blue), as well as mAbs that were ELISA-positive but non-reactive with the EpCAM native form (sky blue), are indicated. Reactivity of the native form of EpCAM was determined by immunocytochemistry. (**c**) Summary of epitope mapping by western blotting. The epitope distribution of the mAbs against the EpCAM native form is indicated.

EpCAM is proteolytically cleaved at multiple sites, especially at Arg^80^–Arg^81^^12,13^; thus, the location of mAb binding is important in the development of therapeutic mAbs. Among the anti-EpCAM mAbs that have been clinically studied, edrecolomab (17-1A) and 323/A3 are mouse-derived IgG2a^14^ subclasses, ING-1 and 3622W94 are human-engineered and humanized mAbs^15^, and all mAbs bind to the N-terminal domain (ND; amino acids 24–62) of human EpCAM^6,16^. Anti-EpCAM mAbs, including AUA-1, Ber-EP4, HEA125, MOC31, and VU-1D9 also bind to ND. Thus, the majority of mAbs that recognize EpCAM on cancer cells bind specifically to the EpCL domain (amino acids 24–80) of EpCAM (Fig. 1a), and that region is considered to offer high immunogenicity^12,17^.

MT201 (adecatumumab), NM104, and 311/1K bind to the cysteine-poor region (amino acids 136–264) of EpCAM, and HO-3 and EpAb2-6 antibodies bind to the thyroglobulin type-1 domain (amino acids 63–135)^6,18^. Adecatumumab, which is a fully human antibody, was isolated from a human IgD-positive B-cell repertoire via guided selection, phage display, and combination with human IgG1 constant domains^19^. EpAb2-6—a mouse antibody—is the first antibody class compound that induces apoptosis by directly inhibiting EpCAM signaling rather than requiring accessory immune mechanisms for cell killing^20^. These mAbs are predicted to be advantageous therapeutically because they bind to the remaining membrane-side region of EpCAM after cleavage at Arg^80^–Arg^81^. Catumaxomab is a bispecific mAb consisting of paired mouse anti-CD3 and rat anti-EpCAM mAbs, and it has been approved for the treatment of malignant ascites^21^. These mAbs mainly function as therapeutics through a mechanism dependent on effector cells of the immune system or a mechanism independent of immune cells that requires circulating proteins of the complement system^22^.

Therapeutic activities have been confirmed in the clinical use of anti-EpCAM mAbs without concern for pancreatic toxicity; however, no objective tumor regression was observed^23,24^. Therefore, enhancement of the anti-tumor effects of EpCAM-directed antibody therapy is needed. To date, several hundred papers have described the structural characterization and analysis of mAb derivatives^25,26^. For instance, oportuzumab monatox (a single-chain variable fragment antibody conjugated with *Pseudomonas* exotoxin A), citatuzumab bogatox (an antigen-binding fragment with bouganin), and the immuno-conjugate antibody tucotuzumab (an antibody conjugated with interleukin-2) have been studied in clinical trials^27^. Conjugating the anti-EpCAM mAb with α-amanitin, which is a toxin that inhibits DNA transcription, resulted in significantly reduced cell proliferation in human pancreatic, colorectal, breast, and bile duct cancer cell lines^24^.

We previously reported that a large number of anti-EpCAM fully human mAbs had been successfully established from TC-mAb mice^4^. Therefore, in this study, we further analyzed their properties, including their ability to bind to native EpCAM on the cell surface and their biological activity as antibody–drug conjugate (ADC) therapeutic agents. Analysis of the mAbs by enzyme-linked immunosorbent assay (ELISA) and western blotting (WB) using truncated EpCAM recombinant proteins in combination with immunocytochemistry (ICC) revealed that a variety of mAbs were induced in TC-mAb mice. Furthermore, the internalization capability of the selected mAbs was determined by ICC internalization assay and directly labeling the mAbs with a maytansine derivative using the chemical conjugation by affinity peptide (CCAP) method^28^. Not only high- but also low-affinity anti-EpCAM fully human mAbs showed clear cytotoxicity against the human colon cancer cell line HCT116, which is a well-known EpCAM-expressing line. Since low-affinity mAbs have been reported to have the potential to penetrate more deeply into solid tumors^29^, a simple CCAP labeling method can provide a powerful tool for the future development of fully human ADC therapeutics. Overall, this study demonstrated that TC-mAb mice can produce a wide variety of mAbs to a target protein and found an efficient method to identify ADC therapeutics, which should accelerate the development of various human therapeutics worldwide.

## Results

### Characterization of anti-human EpCAM fully human mAbs obtained from TC-mAb mice

**Figure 2:**
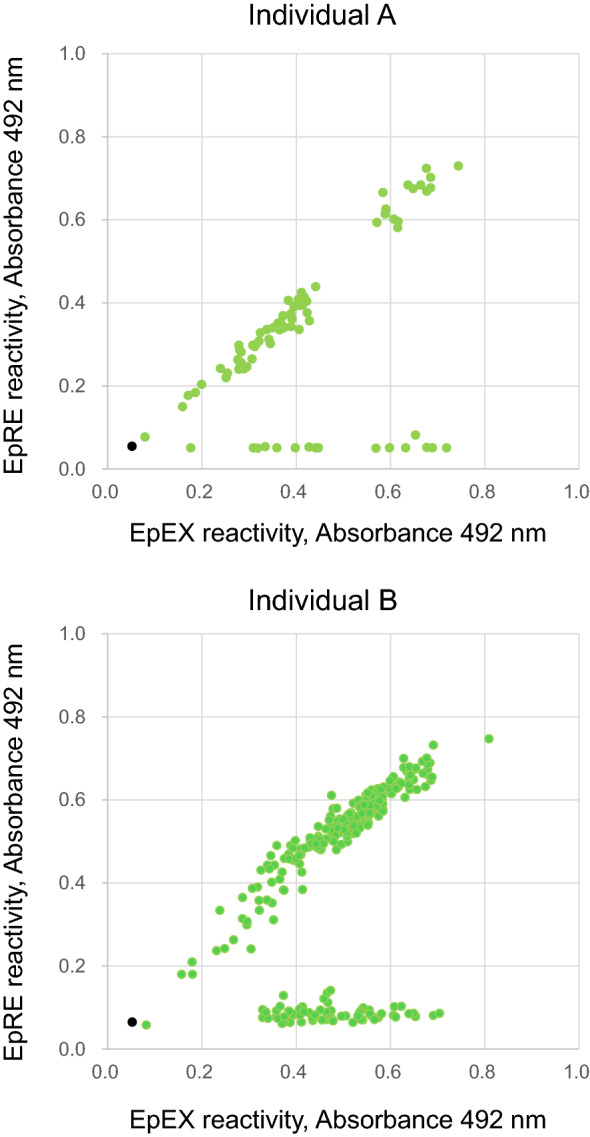
ELISA of monoclonal antibodies. Overall, 80 and 297 mAbs obtained from individuals A (upper) and B (lower) were analyzed by ELISA. The signals of EpEX and EpRE are plotted, showing two groups. The first group that reacts proportionally with the EpEX and the EpRE contains mAbs that react with the EpRE domain of EpCAM. Meanwhile, the other group that reacts with the EpEX but not the EpRE contains mAbs that react with the EpCL domain. Therefore, the first group can be defined as the EpRE-reactive mAbs and the second group as the EpCL-reactive mAbs. Black dot in each graph indicates the value for the negative control value obtained using the medium without mAb.

Previously, we established 377 clones of anti-human EpCAM fully human mAbs from TC-mAb mice^4^. We further analyzed their characteristics in this study. First, the obtained mAbs were classified as EpCL (EpCAM cleaved domain, amino acids 24–80 of EpCAM)- or EpRE (EpCAM membrane residual domain, amino acids 81–265)-reactive mAbs because EpCAM is cleaved at Arg^80^–Arg^81^ on the cell surface^6^. By enzyme-linked immunosorbent assay (ELISA) using recombinant proteins of EpEX (amino acids 24–265) and EpRE, the mAbs were divided into 83 (22.0%) EpCL-reactive and 293 (77.7%) EpRE-reactive mAbs, respectively (Table 1, Fig. 1b, and 2). These values were closely correlated with the molecular weights of EpCL (6.2 kDa, 22.6%) and EpRE (21.2 kDa, 77.4%), indicating that antibody immunogenicity in TC-mAb mice was similar between the EpCL and EpRE regions.

**Table 1:** Summary of analyzed clones.

	TC-mAb mice		
	Individual A	Individual B	Total
Epitope mapping of ELISA-positive clones (clones)			
ELISA-positive (clones)^a^	80	297	377
EpCL	16	67	83
EpRE	64	229	293
Unanalyzed	0	1	1
ICC-positive (clones)^a^	20	52	72
EpCL	9	46	55
EpRE	11	5	16
Unanalyzed	0	1	1

^a^The total numbers of ELISA-positive and ICC-positive clones were already reported in our previous study^4^.

**Figure 3:**
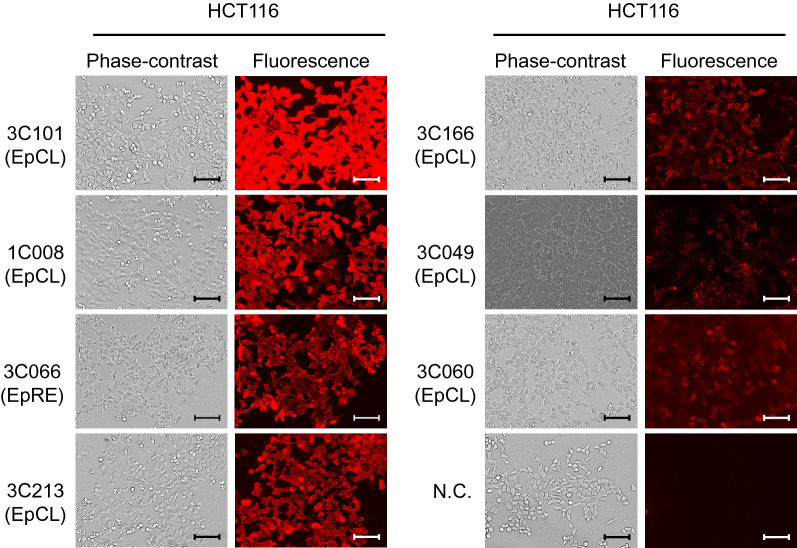
Assessment of anti-epithelial adhesion molecule (EpCAM) fully human monoclonal antibodies. Immunocytochemistry (ICC) of selected mAbs against unfixed HCT116 cells. HCT116 cells were stained with 5 μg/mL mAb. Scale bar = 100 μm. Magnification × 40. The clone number of the analyzed mAb is indicated in the upper left of each figure, and the area of the EpCAM extracellular domain recognized by the mAb is shown in parentheses.

**Figure 4:**
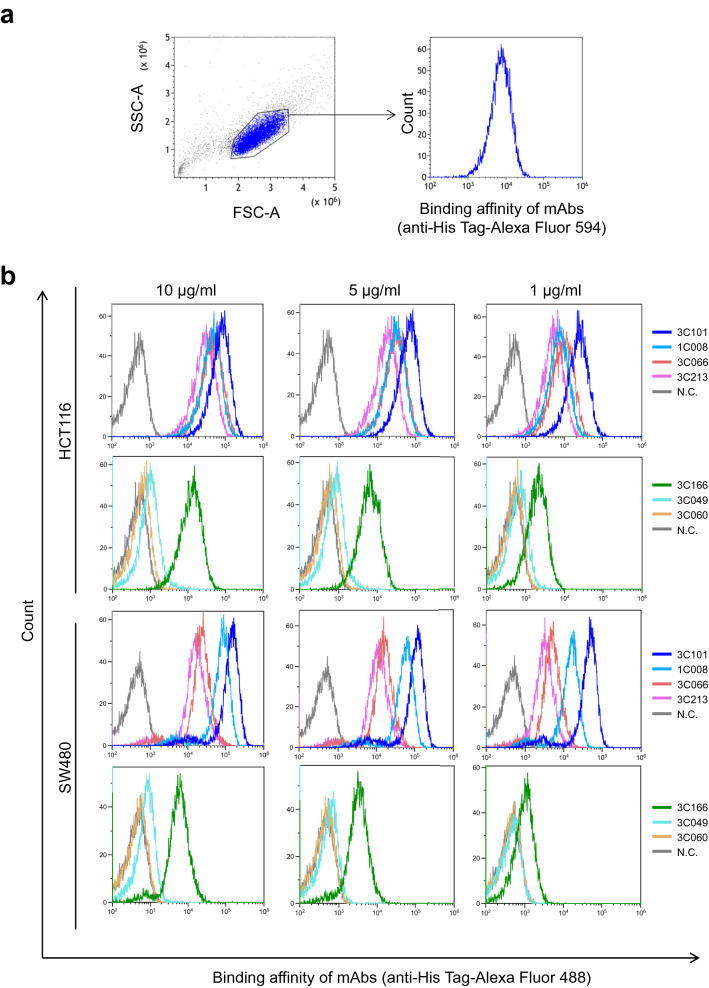
Flow cytometry of monoclonal antibodies. Flow cytometry of mAbs against HCT116 and SW480 cells. (**a**) Gating strategy used in this study. Data are presented in the scatter plot of forward scatter (FSC-A) and side scatter (SSC-A), as well as the histogram indicating Alexa Fluor 488-induced fluorescence intensity. (**b**) Flow cytometry of the selected mAbs. HCT116 and SW480 cells were stained with fully human mAbs at the indicated concentration. Negative control (gray color) indicates the signal with secondary antibody but without primary mAb.

For the development of therapeutic mAbs, their reactivity to the native form of EpCAM on the cell surface is important. Therefore, we employed ICC analysis using unfixed HCT116 and SW480 cells, which are known as EpCAM expressing cell lines. The results allowed us to identify a total of 72 clones, 20 from individual A and 52 from individual B, as reported in our previous study (Table 1, Fig. 3, and Supplementary Fig. 1)^4^. We selected six mAbs with different affinity to EpCAM on the cell surface by ICC. The mAb reactivities with EpCAM were also examined by flow cytometry (FCM) (Fig. 4). The results indicated that ICC staining intensity had some correlation with FCM signal intensity, with 3C101 having the highest affinity; 1C008, 3C066, and 3C213 having moderate and similar affinity; and 3C166, 3C049, and 3C060 having low affinity. However, the results of binding analysis by ELISA using the mAbs were different from the affinity of ICC and FCM, with 3C049 and 3C060 having strong affinity to EpEX protein (Supplementary Fig. 2). This is possibly due to the difference in protein folding of EpEX from the native form of EpCAM and the effect of the fixation of EpEX to the ELISA plate surface.

Taking the findings together, the distribution of epitopes between the EpCL and EpRE regions indicated that 55 of 83 (66.3%) EpCL-reactive mAbs and 16 of 293 (5.5%) EpRE-reactive mAbs could bind to the EpCAM native form on the cell surface, suggesting that the EpCL domain of the EpEX recombinant protein more efficiently induced mAbs that bind to conformational epitopes presented on the cell surface. This result is consistent with previous reports indicating that the EpCL domain of EpCAM produced high immunogenicity^12,13,17^.

### Epitope mapping of anti-EpCAM fully human mAbs

Using the 49 well-grown hybridomas producing mAbs that bound to the EpCAM native form, epitope mapping was performed by WB using truncated EpEX proteins (Fig. 1a,c, and Supplementary Fig. 3). As expected, the majority of epitopes targeted amino acids 62–80 of EpCAM (designated Ep62-80 mAbs). mAbs that recognized additional epitopes were also obtained, but mAbs binding to 24–61 and 185–218 of EpCAM were not identified (Fig. 1c and Supplementary Fig. 4). Because WB detects the signal of mAbs that recognize linear epitopes, the signal of mAbs that bound to conformational epitopes was hardly detected. These results at least indicate the linear epitope to which the mAbs can bind. Consistent with this, there were also mAbs that recognized native structures of EpCAM that could not be analyzed by WB (data not shown).

### Identification of internalizing human EpCAM mAbs

**Figure 5:**
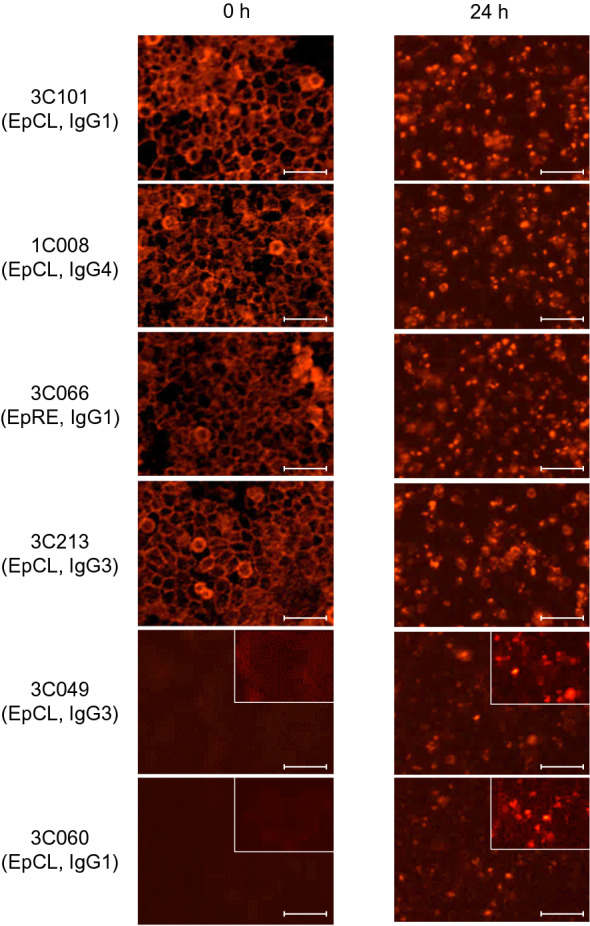
Internalization analysis of anti-EpCAM fully human mAbs. HCT116 cells were stained with the indicated mAbs followed by anti-human IgG-Fc antibody conjugated with Alexa Fluor 594 (The Jackson Laboratory, Bar Harbor, ME, USA) at a dilution of 1:600 and analyzed using IncuCyte S3 (Sartorius, Göttingen, Germany). Fluorescent images taken immediately after the start of incubation at 37 °C for 0 h (left side) and after 24 h of incubation (right side) are shown. Scale bar = 50 μm. Magnification × 40.

According to the staining intensity of ICC and FCM, strongly binding clones (3C101), a moderately binding clone (1C008, 3C066, and 3C213), and weakly binding clones (3C049 and 3C060) were selected for further analysis. Regarding the mAb amino acid sequences of these six mAbs, all mAbs have different L chain variable regions, although 3C060 and 3C049 have the same H chain variable region, confirming that different mAbs were evaluated in this study (Supplementary Fig. 5). Time-lapse imaging analysis showed that internalization into HCT116 cells was clearly detectable for all mAbs analyzed by ICC, regardless of their binding affinity (Fig. 5).

### Cytotoxicity of antibody–drug conjugates

**Figure 6:**
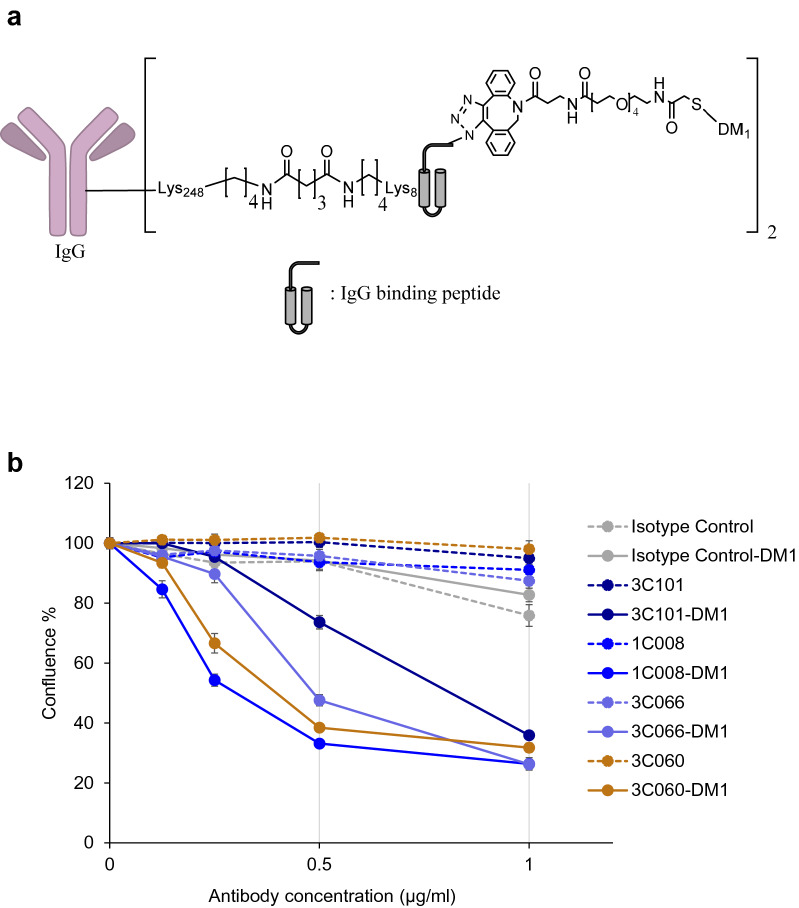
Cytotoxicity of antibody–drug conjugate. (**a**) Schematic design of mAb-IgG-BP-DM1. The selected monoclonal antibody (mAb) was modified with azide-attached chemical conjugation by affinity peptide reagent and labeled with mertansine (DM1) via mixing with DBCO-PEG4-DM1. (**b**) Cytotoxicity of anti-EpCAM mAbs directly labeled with DM1 using the CCAP method. The growth of HCT116 cells was monitored as the percentage confluence. The percentages at 60 h are indicated (n = 3). Data are presented as the mean ± SE.

We further produced and analyzed mAbs that were directly labeled by the anticancer drug DM1 via the affinity peptide CCAP-labeling method (Fig. 6a,b), which easily enabled site-specific modification of anti-human IgGs^28^. Because IgG-BP-CCAP was reported to be a reagent able to modify human IgG1, IgG2, and IgG4, but not IgG3, we selected four clones, namely, 3C101 (IgG1), 1C008 (IgG4), 3C066 (IgG1), and 3C060 (IgG1), with different binding affinity with EpCAM on the cell surface (Fig. 4). IgG was modified with the azide-attached IgG-BP-CCAP reagent and labeled with DM1 via mixing with DBCO-PEG4-DM1 through click chemistry. A schematic view of DM1-labeled IgG is presented in Fig. 6a. According to SDS-PAGE, approximately 50%–70% of the protein of each mAb was estimated to be labeled by one or two molecules of DM1 (Supplementary Fig. 6). Nearly all of the isotype control IgG1 was labeled by two molecules of DM1. The results indicated that all directly labeled anti-EpCAM but not the isotype control human IgG1 displayed clear cytotoxicity at protein conjugate concentrations of 0.25–1 µg/mL (Fig. 6b and Supplementary Fig. 7). The 50% inhibitory concentrations (IC_50_) were calculated as 0.553, 0.208, 0.402, and 0.254 μg/mL for 3C101-, 1C008-, 3C066-, and 3C060-DM1 conjugates, respectively, assuming that all HCT116 cells were killed at the concentration of 1 μg/mL. The 1C008-DM1 conjugate displayed the greatest cytotoxicity, and 3C060-DM1 and 3C066-DM1 also exhibited significant cytotoxicity. However, the cytotoxicity of the CCAP-DM1-conjugate with 3C101 mAb, which showed the highest staining intensity by ICC and FCM (Figs. 3, 4), was weaker than that of the other mAb-CCAP-DM1 conjugates. Thus, the cytotoxicity of mAb-CCAP-DM1 conjugate is not significantly correlated with the binding affinity determined by fluorescence-labeled secondary Abs.

This study found that the CCAP direct labeling method can detect not only high-affinity mAbs such as 1C008 but even low-affinity ones such as 3C060, which is difficult to detect with FCM and ICC. Notably, 3C066 mAb with ADC activity was obtained among EpRE-reactive antibodies, for which difficulty in producing anti-EpCAM antibodies has been reported.

## Discussion

We previously reported that a large number of anti-EpCAM fully human mAbs were efficiently established from TC-mAb mice. In this study, we further characterized these mAbs by detailed analysis of epitope mapping (Fig. 1 and Supplementary Figs. 3, 4) in combination with native structure recognition of mAbs (Figs. 3, 4, and Supplementary Fig. 1). EpCAM is cleaved at Arg^80^–Arg^81^ on the cell surface^6^, and it is also difficult to produce anti-EpCAM mAbs that react with the EpRE domain, which is an excellent target for antibody drugs left on the membrane side^12,13^. Consistent with this, the epitope distribution of mAbs reacting with the native form of EpCAM is dominated by EpCL (amino acid 24–80)-reactive mAbs, compared with EpRE (amino acid 81–265)-reactive mAbs (Fig. 1b). Because the mAbs reacting with the EpCAM cellular region (EpEX) by ELISA are proportional to the molecular weights of EpCL and EpRE, the antigenic region of the protein sequence is considered to be widespread throughout the entire EpEX protein. The lower production efficiency of EpRE-reactive mAbs is possibly attributable to the structural similarities between the human and mouse EpRE domains, epitope hiding by dimerization, and glycoside modification of the protein on the cell surface^6^.

To screen ADC therapeutics, we confirmed that the CCAP-labeling method revealed that all internalizing mAbs analyzed exhibited significant cytotoxicity (Fig. 6b). However, we detected strong cytotoxicity of the isotype control human IgG1–DM1 conjugate at concentrations exceeding 2.5 µg/mL (Supplementary Fig. 8). In our experience, the supernatant obtained from hybridoma cell culture contains human IgG at concentrations of 0.1–100 µg/mL (mean ~ 30 µg/mL). If all human IgG proteins in the supernatant were labeled with IgG-BP-CCAP, then some of the nonspecific mAbs may induce cytotoxicity. Thus, it is necessary to use a low concentration of IgG-BP-CCAP reagent (≤ 1 μg/mL) or further develop a screening molecule for ADC therapeutics such as fusion molecules containing IgG-BP-CCAP and low-molecular-weight toxins that do not exhibit any cytotoxicity in supernatant containing nonspecific human IgG at concentrations exceeding 30 µg/mL.

Among the selected four clones for the analysis of ADC activity, there are differences in the order of binding affinity for HCT116 cells (3C101 > 1C008 > 3C066 > 3C060) (Fig. 4) and ADC cytotoxicity on HCT116 cells (1C008 > 3C060 > 3C066 > 3C101) (Fig. 6). The reason for this remains to be elucidated, but in the CCAP method, up to two molecules of DM1 are covalently labeled per antibody in the Fc region of the antibody, which minimizes antibody inactivation during the labeling process and variations of cross-linking payload between antibody types. Since the CCAP method does not interfere with the binding ability of the antibody itself^28^, and the reason 3C101-DM1 did not show the highest cytotoxicity is possibly due to low labeling efficiency. When a linker sequence that is easily cleaved intracellularly is inserted between mAb and the drug molecule, ADC activity may be detected that correlates with the strength of binding of the mAb to the cell surface antigen. We found that the 3C060 mAb was a weak-affinity mAb against HCT116 cells, according to ICC and FCM (Figs. 3, 4), but it displayed clear cytotoxicity in the CCAP method (Fig. 6). Because mAbs with lower binding affinity may penetrate deeply into solid tumors^29^, these low-affinity internalizing mAbs should not be ignored and this is a possible advantage of the CCAP method. Taking the findings together, we demonstrated that fully human mAbs are successfully obtained from TC-mAb mice and screened by using the IgG-BP CCAP, thereby effectively identifying putative candidates such as 1C008 (EpCL-reactive mAb) and 3C066 (EpRE-reactive mAb) for fully human ADC therapeutics.

To address the efficiency of internalizing mAb identification, an antibody-labeling molecule has the advantage of being available for screening with supernatant containing antibodies without determining the antibody concentrations, and it can also be used for mAbs with unknown antigens that are induced by cell administration^30,31^. Therefore, when probes with low cytotoxicity after cellular internalization are obtained by the CCAP method, it may be possible to efficiently identify fully human mAbs for subsequent development as therapeutic mAbs for ADCs. While this study focused on establishment of a screening method, a challenging issue of ADCs losing FcRn binding needs to be addressed for further investigation as therapeutic ADCs through, for instance, eluting the purified monovalent antibody with mild acidic buffer^32^.

The findings in this study show that mAbs against membrane protein, EpCAM, can be efficiently obtained from TC-mAb mice and that the CCAP method efficiently identifies internalized mAbs, which should accelerate the development of various human therapeutics worldwide.

## Methods

### Materials

HCT116 (CCL-247) and SW480 cells (CCL-228) were purchased from the American Type Culture Collection (Manassas, VA, USA). The cells were maintained at 37 °C in an atmosphere of 5% CO_2_. Restriction enzymes and DNA-modifying enzymes were purchased from New England Biolabs (Ipswich, MA, USA) and TOYOBO (Tokyo, Japan), respectively. Primers and DNA fragments were synthesized by Eurofins (Huntsville, AL, USA) or Thermo Fisher Scientific (Waltham, MA, USA). *Escherichia coli* strains [DH5α and Rossetta-gamiB pLysS(DE3)] were purchased from Takara Bio (Kusatsu, Japan) and Merck Millipore (Billerica, MA, USA). Reagents were purchased from Wako Pure Chemicals (Osaka, Japan) or Sigma-Aldrich (St. Louis, MO, USA), unless otherwise stated.

### Preparation of antigens

To generate the recombinant proteins, the extracellular domain of human EpCAM (NM_002354) was fused with Trx-Tag (designated Trx-EpEX or EpEX) or GST-Tag (GST-EpEX), as reported previously^4^. The recombinant proteins of EpRE, EpN62, EpC162, EpC184, and EpC218 (Fig. 1a) were also obtained using the same method. In brief, DNA fragments were amplified by PCR using each primer set (Supplementary Table 1) and subcloned into pET32b (Merck Millipore) using *Eco*RV and *Hind*III (resulting in EpEX). To obtain the GST-EpEX recombinant fusion protein, the fragment was subcloned into pGEX6P1 (GE Healthcare, Chicago, IL, USA) using *Bgl*II and *Not*I. After transformation of *E. coli* Rossetta-gami B pLysS (DE3) with each vector, the recombinant proteins were expressed by induction with 1.0 mM isopropyl-β-d-(−)-thiogalactopyranoside in LB medium. After harvesting and sonicating the cells, the recombinant proteins were purified using Ni–NTA agarose (QIAGEN, Venlo, Netherlands) and dialyzed against PBS containing 0.4 M arginine. The recombinant protein samples were diluted to approximately 1 mg/mL and stored at − 30 °C.

### ELISA

The antibody titers were measured using the same ELISA method as in our previous study^4^. In brief, 96-well immunoassay plates (Nunc Maxisorp, Thermo Fisher Scientific) were coated with 100 µL/well of antigen overnight, and blocked with PBS containing 5% skimmed milk (Difco) for 30 min at room temperature. After washing, 100 µL antibody samples were added to wells and incubated for 1 h at room temperature. The plates were washed again and incubated with 100 µL of goat anti-Human IgG (H + L) Cross-Adsorbed Secondary Ab (Abcam, Cambridge, UK) at 1/50,000 dilution in TBS-T for 30 min at room temperature. The plates were washed once again and developed using 100 µL of *o*-phenylenediamine dihydrochloride, with the reaction being stopped using 25 µL of 1 M H_2_SO_4_. After developing for 15 min, absorbance was read at 492 nm.

### Cell staining

Cells cultured on a 96-well plate or glass-bottomed plate (Matsunami, Osaka, Japan) were stained with 100 µL of supernatant or purified mAb followed by incubation for 1 h on ice. After washing with ice-cold medium, 100 µL of goat anti-Human IgG (H + L) Cross-Adsorbed Secondary Antibody, Alexa Fluor 488, or Alexa Fluor 594 (Abcam, Cambridge, UK) diluted 400-fold in medium was added. After additional washing, 100 µL of PBS with 1% v/v fetal bovine serum (FBS) was added for scanning via fluorescence microscopy (BZ-X700; Keyence, Itasca, IL, USA). For antibody internalization analysis, stained plates were scanned using a live-cell analysis system (IncuCyte S3; Sartorius, Göttingen, Germany). For FCM, cultured cells were dissociated using Accutase (Innovative Cell Technologies, Inc., San Diego, CA, USA) and stained. Stained cells were analyzed using a CytoFLEX S (Beckman Coulter, Brea, CA, USA). All staining reactions were incubated at 4 °C for 30 min using 1 × 10^6^ cells in 100 µL of staining buffer (PBS with 5% FBS:BD Biosciences Brilliant stain buffer, 1:1; BD Biosciences, Franklin Lakes, NJ, USA).

### Western blot

Whole-cell extracts were prepared from sub-confluent cell cultures using RIPA buffer [0.1% (w/v) SDS, 0.5% (w/v) deoxycholate, 1% (v/v) NP-40, 150 mM NaCl, 50 mM Tris–Cl (pH 8.0)]. The extracts or purified antigen were subjected to SDS-PAGE and transferred to PVDF membranes (Bio-Rad Laboratories Inc., Hercules, CA, USA). The membranes were blocked [5% (w/v) skim milk] and incubated with fully human mAbs followed by incubation with anti-human IgG-Fc HRP-conjugated secondary antibody (Bethyl Laboratories). Antibody complexes were reacted with the Clarity Western ECL Substrate (Bio-Rad Laboratories Inc.) and visualized using the LAS4000 mini system (GE Healthcare).

### Direct labeling of mAbs

The IgG-BP-N3RRR peptide {acetyl-[Lys(Azide)]RRRGSGPDCAYHKGELVWCTFH-CONH_2_, the Cys residues of which were intramolecularly crosslinked by disulfide bonds} was chemically synthesized and purified by Eurofins. The peptide was then modified using disuccinimidyl glutarate, in accordance with a previous report^28^. CCAP reagent (IgG-BP-N3RRR-SG) was solubilized with DMSO at 1 mM. A tenfold molar excess of CCAP reagent was added to the mAb solutions (3–8 µM IgG in PBS buffer). After incubation at room temperature for 30 min, the reaction mixture was dialyzed against PBS to remove the excess CCAP reagent. The modified mAb with IgG-BP-N3RRR-SG was mixed with a three-fold molar excess of DBCO-PEG4-DM1, which was synthesized by mixing bromoacetyl-PEG4-amino-DBCO (Quanta) and DM1 (MedChemExpress) at equimolar amounts in a solution of 10% 0.1 M NaHCO_3_ and 90% DMSO, and incubated for 2 h. The labeled protein was applied to a Sephadex-50 column to remove low-molecular-weight drugs and peptides^28^ and concentrated under a vacuum using a centrifugal evaporator (Eyela CVE-3000).

### Cytotoxicity assay

To analyze the cytotoxicity, HCT116 cells were seeded at 5.0 × 10^3^ cells/well in 96-well plates and incubated with mAbs or mAb–DM1 conjugates for 60 h. The growing cells were monitored by IncuCyte S3. The growth inhibition was detected as the area of adherent cells, indicating percent phase object confluency.

### Statistical analysis

For analyses of the cytotoxicity of ADC complexes comprising anti-EpCAM mAbs, an unpaired Student’s *t*-test was performed. *P* < 0.01 indicated statistical significance.

## Supplementary Information

Supplementary Information 1.

Supplementary Information 2.

## Data Availability

Source data are provided with this article. The DNA sequence of recombinant proteins and antibodies used in this study have been deposited in the DDBJ database under accession numbers LC722463 (EpEX), LC722464 (EpRE), LC722465 (EpN62), LC722466 (EpC162), LC722467 (EpC184), LC722468 (EpC218), LC722469 (3C101 H chain), LC722470 (3C101 L chain), LC722471 (1C008 H chain), LC722472 (1C008 L chain), LC722473 (3C066 H chain), LC722474 (3C066 L chain), LC722475 (3C213 H chain), LC722476 (3C213 L chain), LC722477 (3C166 H chain), LC722478 (3C166 L chain), LC722479 (3C049 H chain), LC722480 (3C049 L chain), LC722481 (3C060 H chain), and LC722482 (3C060 L chain). These sequences can be searched with getentry (http://getentry.ddbj.nig.ac.jp/top-e.html), a database search tool of DDBJ.
