# Supplementary material for: Characterization of human anti-EpCAM antibodies for developing an antibody–drug conjugate
Source: Sci Rep. 2023 Mar 14;13:4225. doi: 10.1038/s41598-023-31263-x (PMC10015092; doi:10.1038/s41598-023-31263-x)
Supplement: Supplementary file 1 — Supplementary Information 1. [file 41598_2023_31263_MOESM1_ESM.pdf]

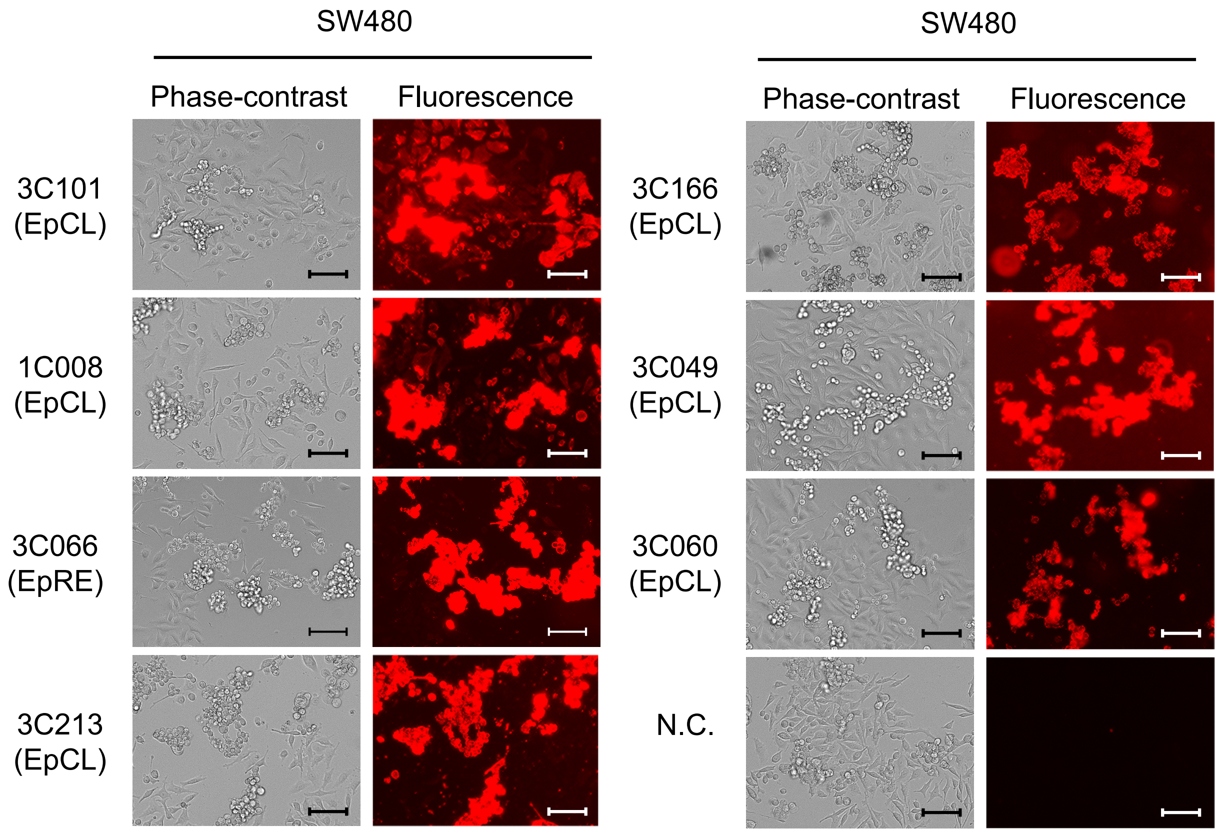

Supplementary Figure 1. Immunocytochemistry of monoclonal antibodies.
Phase-contrast and fluorescence microscopy of SW480 cells stained with the selected mAbs. Each cell line was stained with 10 μg/ml mAb. It has been reported that the SW480 human colon cancer cell line consists of two types [round (R) type and epithelial (E) type] of cells. The R-type cells represent a more malignant variant than the E-type cells [1, 2]. Accordingly, the immunostaining results demonstrated that the signal of the R-type cells was significantly stronger than that of the E-type cells, consistent with the correlation between high EpCAM expression and cancer cell malignancy. Scale bar = 100 μm. Magnification ×40.

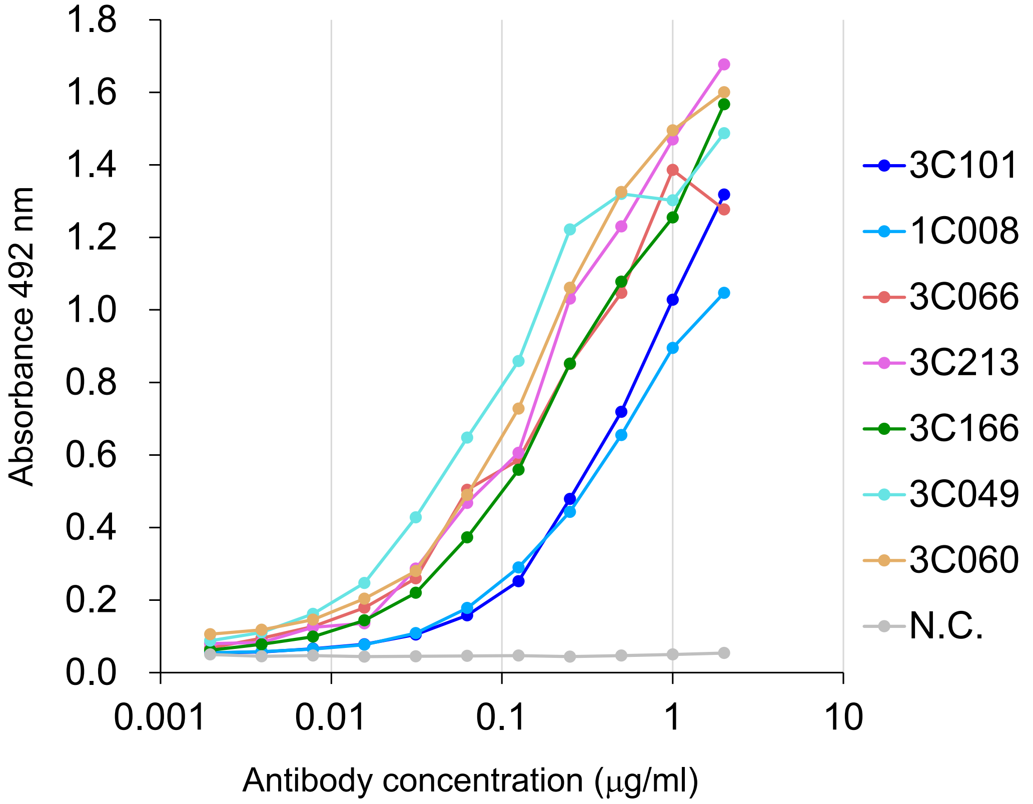

Supplementary Figure 2. ELISA of monoclonal antibodies.

Comparison of mAbs by enzyme-linked immunosorbent assay (ELISA). The mAb was diluted from 2 μg/ml to 1.953 ng/ml by serial dilutions in half. In all figures, N.C. indicates the negative control, in which the cells and plates were stained without primary fully human mAb.

a

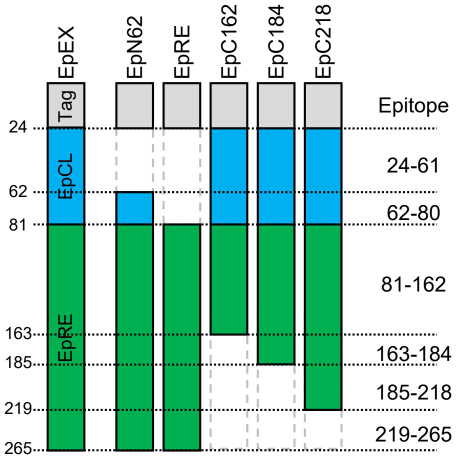

Epitope	EpN62	EpRE	EpC162	EpC184	EpC218
24-61	-	-	+	+	+
62-80	+	-	+	+	+
81-162	+	+	+	+	+
163-184	+	+	-	+	+
185-218	+	+	-	-	+
219-265	+	+	-	-	-

b

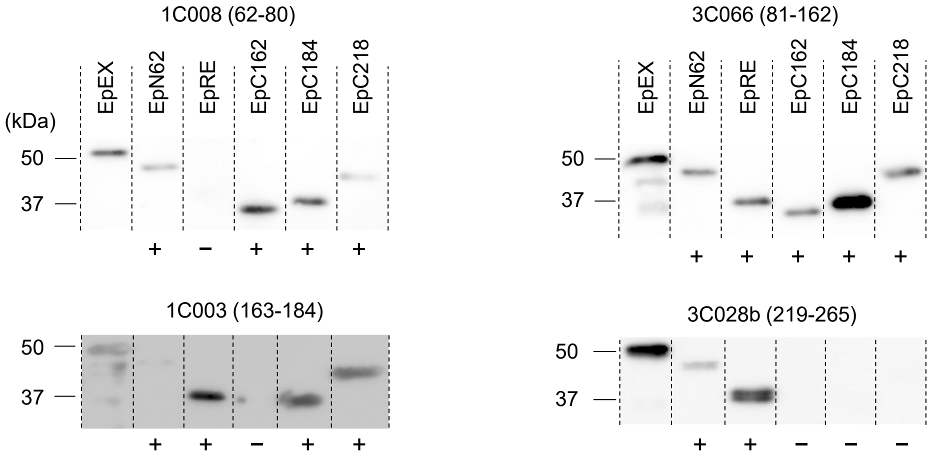

Supplementary Figure 3. Epitope mapping.

(**a**) Schematic diagram of truncated epithelial adhesion molecule (EpCAM). The EpCL region is illustrated in blue, the EpRE region is illustrated in green, and truncated fragments are shown as dotted gray lines. The table presents the reactivity of monoclonal antibodies (mAbs) for the mapped epitope using six recombinant proteins. (**b**) Representative results of western blotting and epitope determination. The truncated proteins treated with the selected mAbs are indicated. Positive (+) signals indicate detection of the recombinant protein.

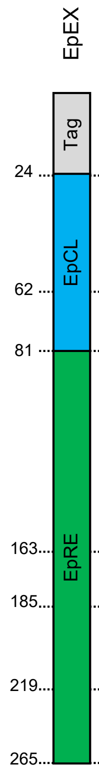

Region	Number of clones: Individual A	Individual B
24-61	0	0
62-80	3	38
81-162	0	4
163-184	1	0
185-218	0	0
219-265	1	2
Total	5	44

Individual A

Clone Number	Epitope
1C008	62-80
1C029	62-80
1C062	62-80
1C003	163-184
1C076	219-265

Individual B

Clone Number	Epitope	Clone Number	Epitope
3C008	62-80	3C169	62-80
3C024	62-80	3C173	62-80
3C028a	62-80	3C194	62-80
3C035	62-80	3C202	62-80
3C044	62-80	3C213	62-80
3C049	62-80	3C214	62-80
3C052	62-80	3C227	62-80
3C058	62-80	3C232	62-80
3C060	62-80	3C248	62-80
3C068	62-80	3C250	62-80
3C076	62-80	3C256	62-80
3C101	62-80	3C260	62-80
3C109	62-80	3C266	62-80
3C124	62-80	3C286	62-80
3C138	62-80	3C287	62-80
3C145	62-80	3C293	62-80
3C146	62-80	3C014	81-162
3C155	62-80	3C066	81-162
3C159	62-80	3C073	81-162
3C163	62-80	3C271	81-162
3C164	62-80	3C028b	219-265
3C166	62-80	3C085	219-265

Supplementary Figure 4. Classification of obtained monoclonal antibodies (mAbs).
The numbers of clones are classified according to the immunoreactive regions of epithelial adhesion molecule. The table summarizes the clone number, subclass, and epitope of each mAb.

H chain

```
                                        CDR1                      CDR2                                            CDR3
3C101H   EVQLVESGGGLVQPGGSLRLSCAASGFTFSSYDM--HWVRQATGKGLEWVSAI-GTVG-DTYYLGSVKGRFTISRENAKNSLYLQMNSLTAGDTAVYYCA---REMANYYYYGMDV
1C008H   ..................................--.................-....-..F.TA.......V................R..........---...G.N.......
3C066H   Q.........V....R................G.--......P.......AV.WYDGS-NK..AD..........D.S..T........R.E........GGEQWSVRG.......
3C213H   ..................................--...............G.-..A.-....P.........................R...........---.............
3C049H   Q...QQ..P...K.SQT.S.T..I..DSV..NSAAWN.I..SPSR....LGRTYYRSKWNND.AA...S.I..NPDTS..QFS..L..V.PE........RREGMGWFDP------
3C060H   Q...QQ..P...K.SQT.S.T..I..DSV..NSAAWN.I..SPSR....LGRTYYRSKWKND.AA...S.I..NPDTS..QFS..L..V.PE........RREGMGWFDP------
```

L chain

```
                                          CDR1                    CDR2                                      CDR3
3C101L   DIQMTQSPSSLSASVGDRVTITCRASQ------GISNYLTWYQQKPGKVPNLLIYAASTLQSGVPSRFSGSGSGTDFTLTISSLQPEDVATYYCQNYNSVPFT
1C008L   E.VL....GT..L.P.E.A.LS.....------SV.H..........QA.R..VFG..IRAT.I.D................R.E...F.L....H.GNS..S
3C066L   .......................Q...------DTR...N........A.K....DV.N.ET...L............F..N......I...F.HQ.YNL.L.
3C213L   ...........................------.......A....Q.....K....G..I....................................S.......
3C049L   .NV.....D..AV.L.E.A..N.KS..TVLYISDNK...A.......QP.K...HW...RK....D...................A....V....Q.YDT.T-
3C060L   .TV.....D..AV.L.E.A..R.KS..SLLYIYDNK..VG.......QP.K....W...RE....D..................EAD...V....Q.YDT.T-
```

Supplementary Figure 5. Sequence alignment of variable regions.
Sequence alignment of the obtained monoclonal antibodies. The amino acid sequences are aligned according to their sequence similarities. Boxes indicate the complementarity-determining region (CDR) of variable regions. The heavy and kappa chain amino acid sequences of 3C101 and 3C213 were already reported in our previous study [4].

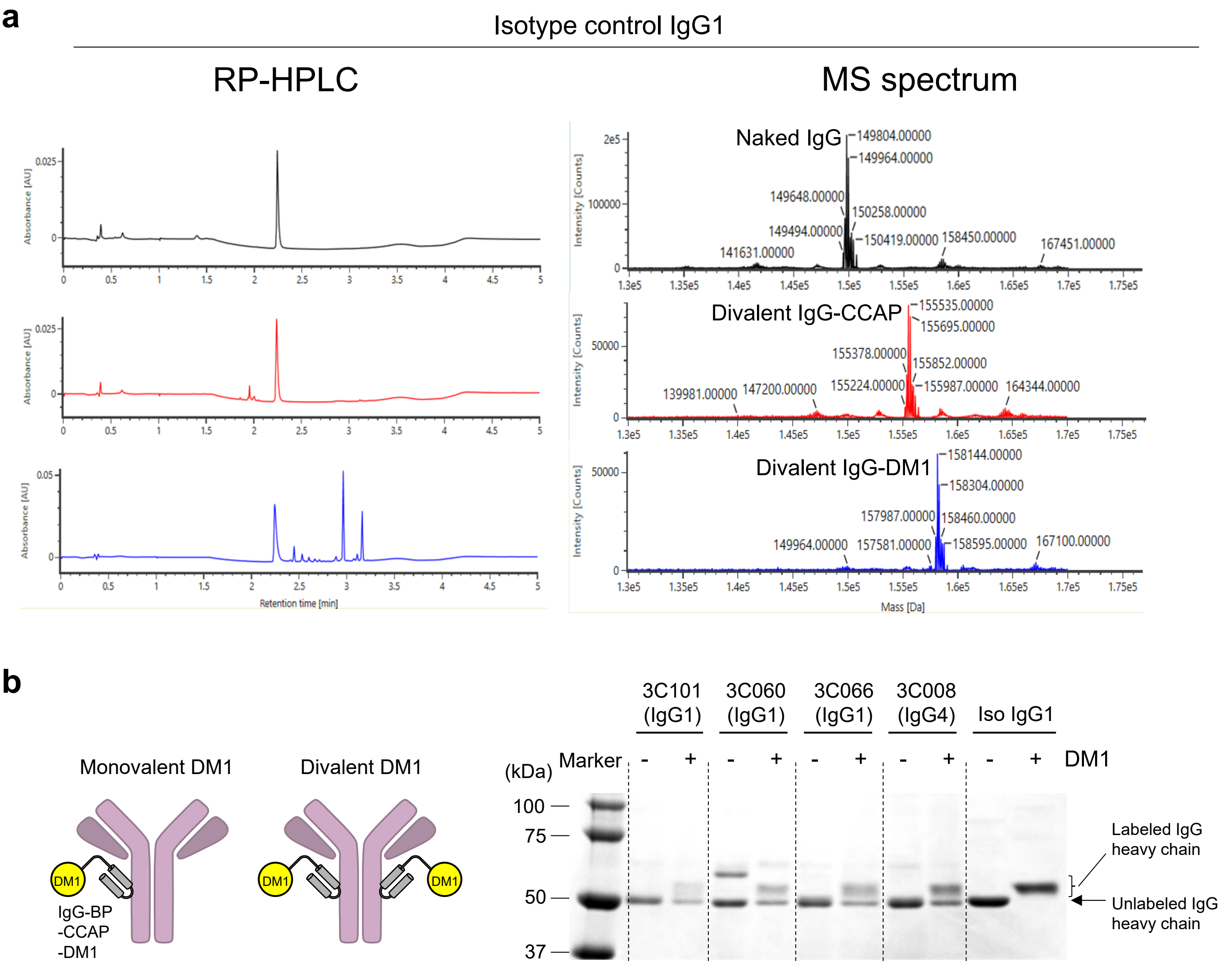

Supplementary Figure 6. Liquid chromatography–mass spectrometry (LC-MS) and SDS-PAGE of directly conjugated monoclonal antibodies (mAbs).

LC-MS analysis of antibodies directly labeled with mertansine (DM1). Antibodies were labeled with chemical conjugation by affinity peptide (CCAP) reagent and subsequently with DM1 for analysis via LC-MS and SDS-PAGE. Typical LC-MS data for modification of isotype control human IgG1 are presented. (**a**) The left and right panels indicate the results for reverse-phase HPLC monitored by the absorbance at 280 nm and mass spectra. The antibody peaks at the elution time of 2.12 min during HPLC were analyzed via MS. Top, middle, and bottom panels indicate the results for unmodified, CCAP reagent-modified, and DM1-conjugated antibodies, respectively. (**b**) SDS-PAGE. Representative images of monovalent and divalent DM1-conjugated mAbs are indicated (left). Unlabeled and labeled mAbs (5 mg/lane) were analyzed by SDS-PAGE (8% gel) under reducing conditions and stained with CBB R-250. Light chains of mAbs (approximately 25 kDa) did not react with CCAP reagent, and they could not be observed in the gel because of their size. The image was captured by Printgraph (ATTO, Tokyo, Japan). The labeling efficiency of each anti-epithelial adhesion molecule mAb was approximately 50%–70%, and that of the isotype control was 100%, as assessed by the signal intensity of the gel and the peak areas. We estimated that the purified mAbs were equally contaminated by bovine IgG, despite the use of Protein G Sepharose to remove the contaminant from FBS in advance.

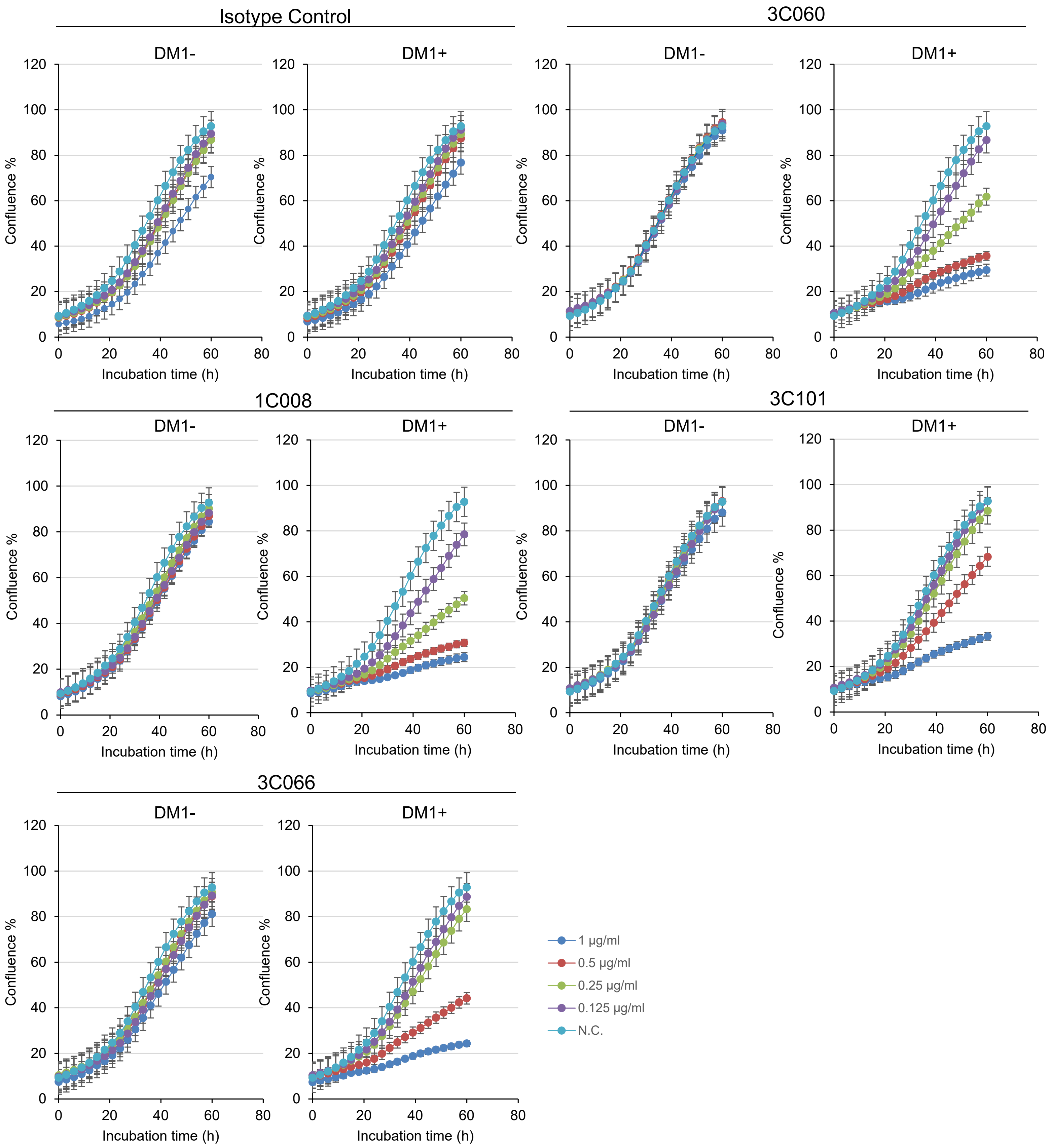

Supplementary Figure 7. Growth curves of HCT116 cells incubated with or without monoclonal antibodies (mAbs) conjugated with mertansine (DM1).

The percent HCT116 cell confluence. Cell confluence was monitored for 60 h. In the negative control (N.C.), HCT116 cells were cultured without any antibodies. The data were collected at 3-h intervals (n = 3). Data represent the mean ± SE.

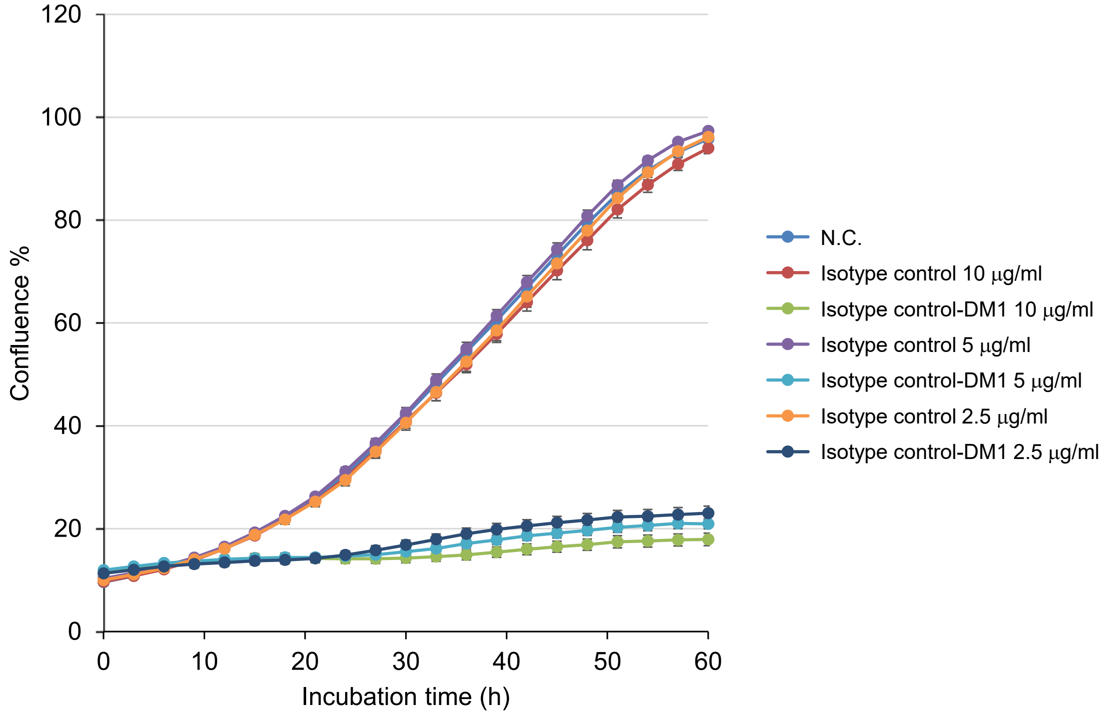

Supplementary Figure 8. Cytotoxicity of isotype control human IgG1–mertansine (DM1) conjugates at a high concentration.

Growth curves of HCT116 cells treated with various concentrations of isotype control human IgG1–DM1 conjugates (2.5, 5.0, and 10.0 mg/ml). In the negative control (N.C.), HCT116 cells were cultured without any antibodies. The percent cell confluence was monitored for 60 h. The data were collected at 3-h intervals (n = 3). Data represent the mean ± SE.

1C008 (24-61)

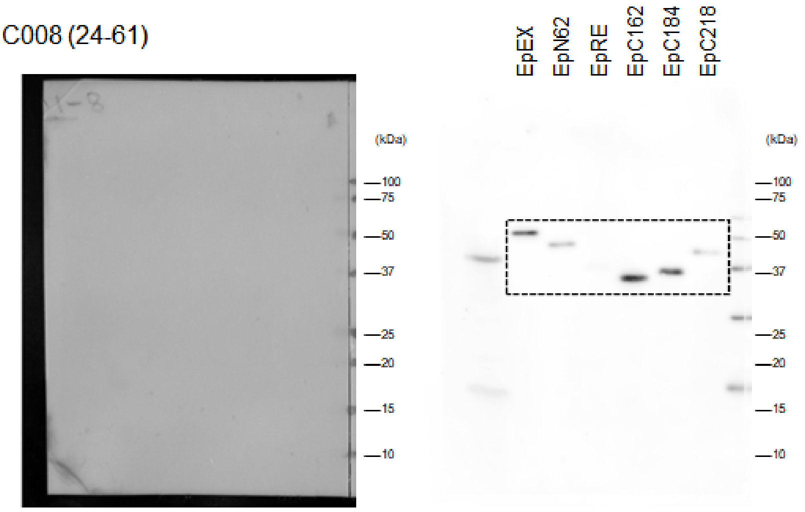

3C066 (81-162)

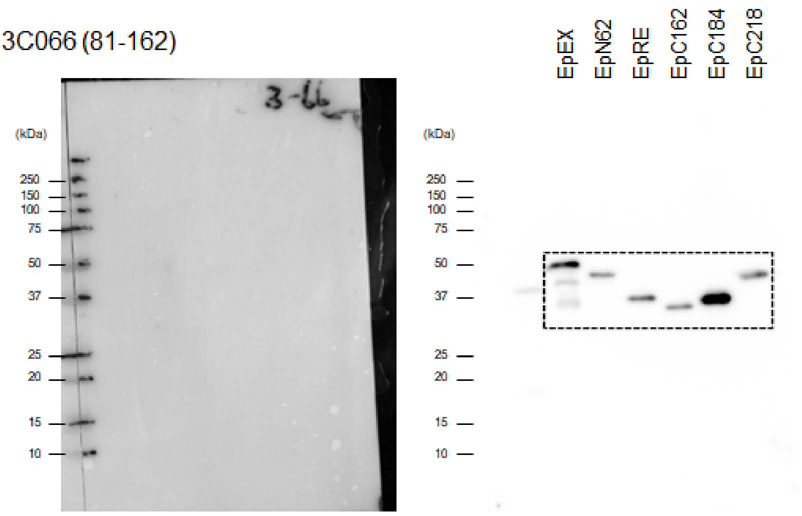

1C003 (163-184)

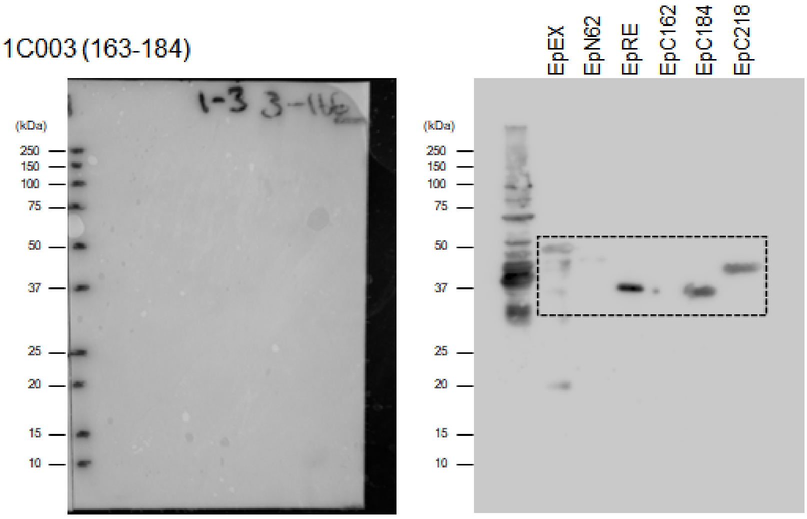

3C028b (219-265)

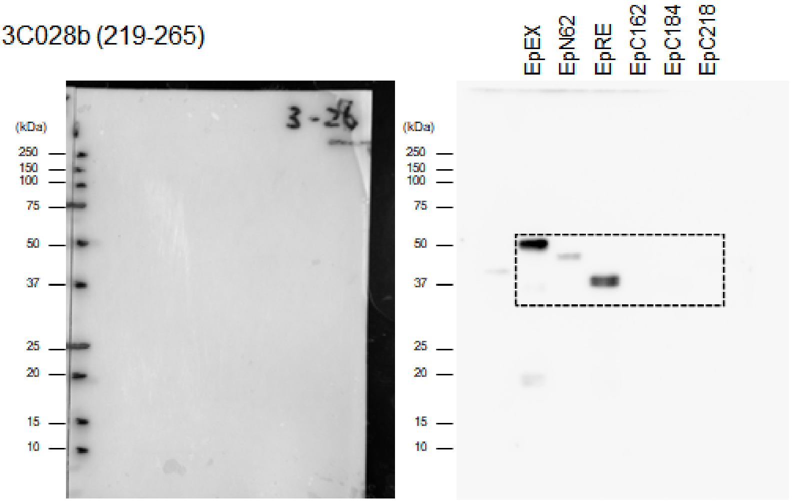

Supplementary Figure 9. Uncropped images of western blotting.
Uncropped images of all WB analyses are presented. The upper left of the figure shows the clone number (binding domain of EpCAM), the left figure shows a visual image of the uncropped membrane, the right figure shows the uncropped WB image, and the dotted square indicates the figure used in Supplementary Fig. 4.

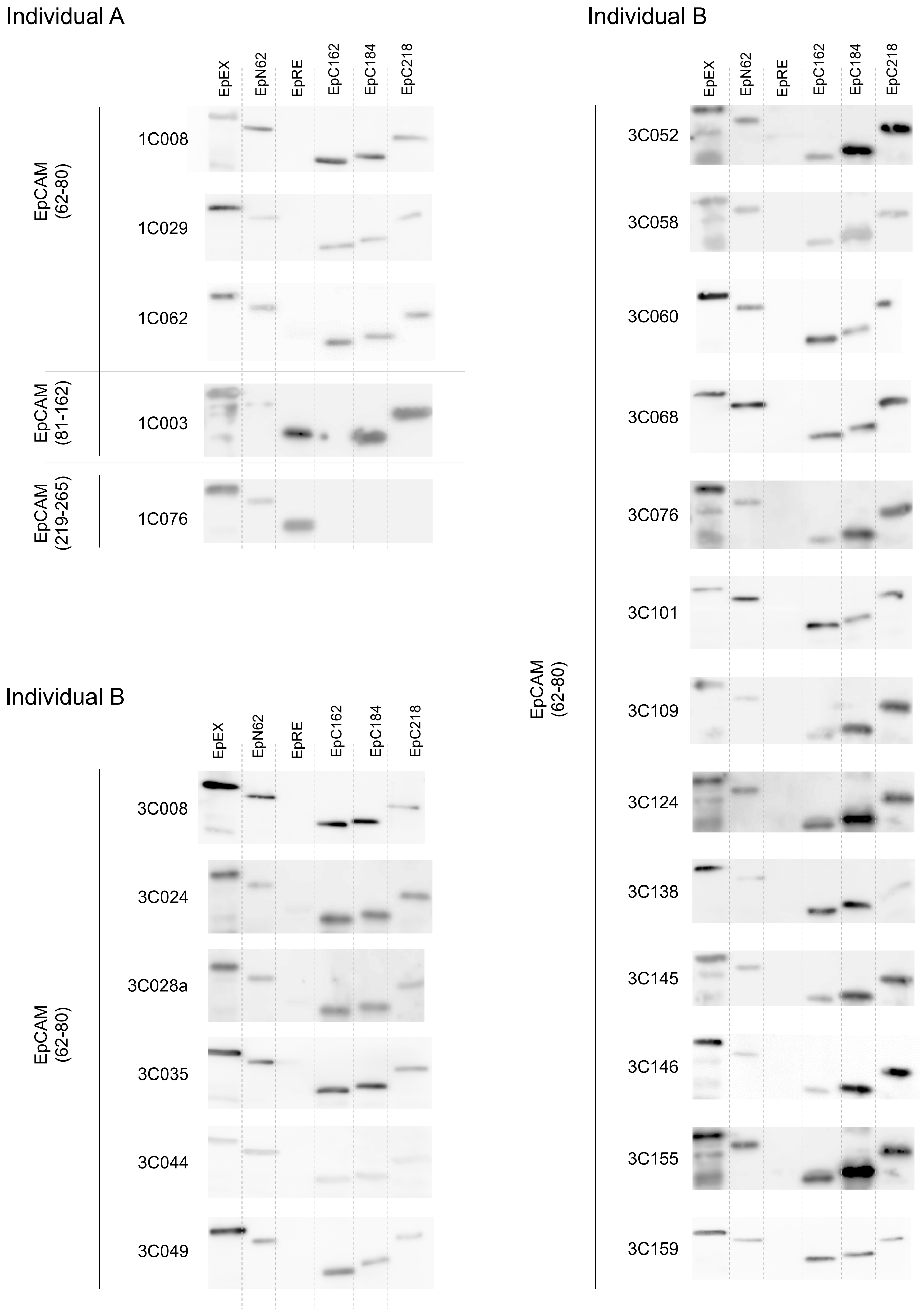
Individual A
EpEX
EpN62
EpRE
EpC162
EpC184
EpC218
EpCAM (62-80)
1C008
1C029
1C062
EpCAM (81-162)
1C003
EpCAM (219-265)
1C076
Individual B
EpEX
EpN62
EpRE
EpC162
EpC184
EpC218
EpCAM (62-80)
3C008
3C024
3C028a
3C035
3C044
3C049
Individual B
EpEX
EpN62
EpRE
EpC162
EpC184
EpC218
EpCAM (62-80)
3C052
3C058
3C060
3C068
3C076
3C101
3C109
3C124
3C138
3C145
3C146
3C155
3C159

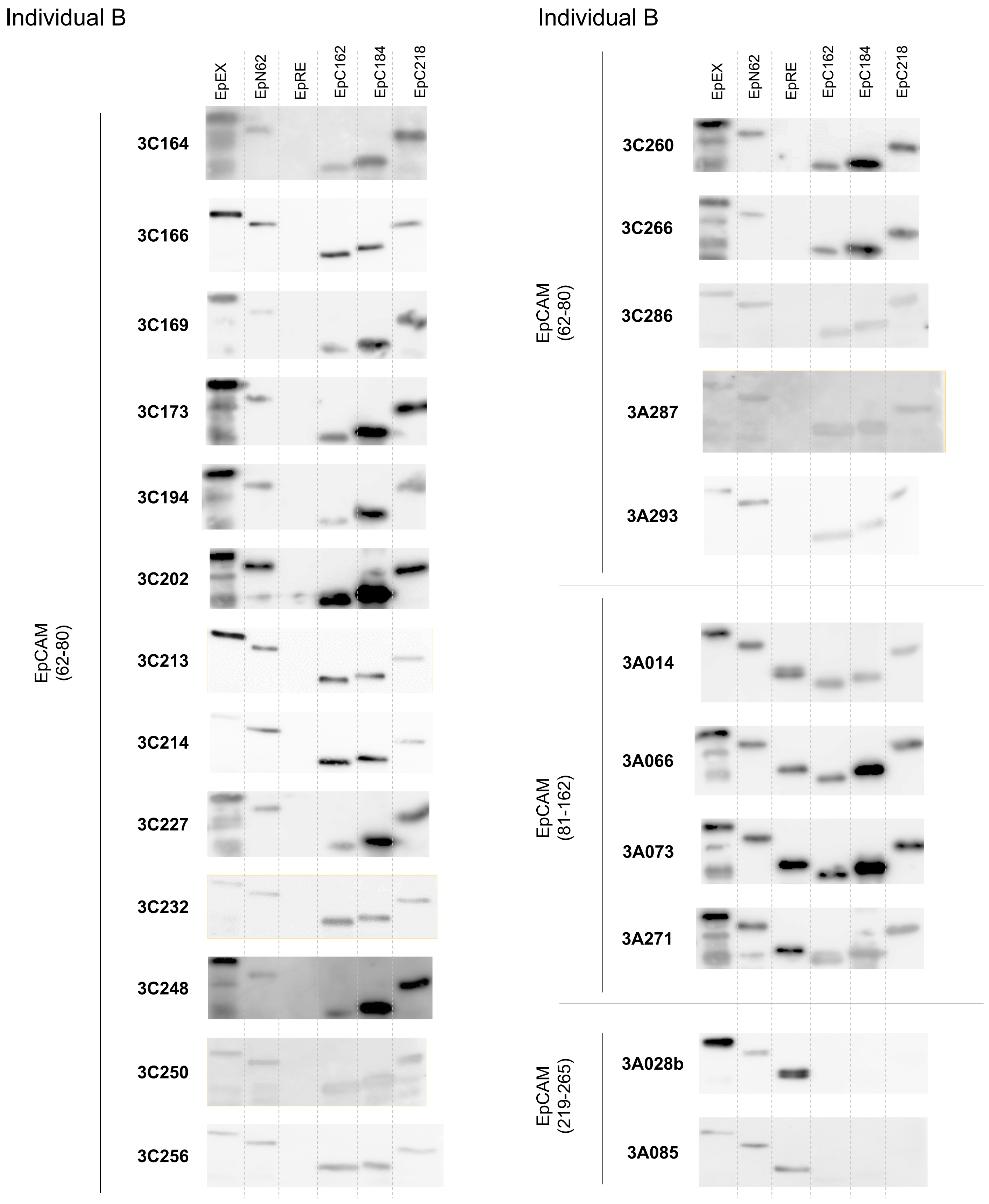

Supplementary Figure 10. Summary of epitope mapping by WB.
The cropped images of epitope mapping by WB analysis are indicated.

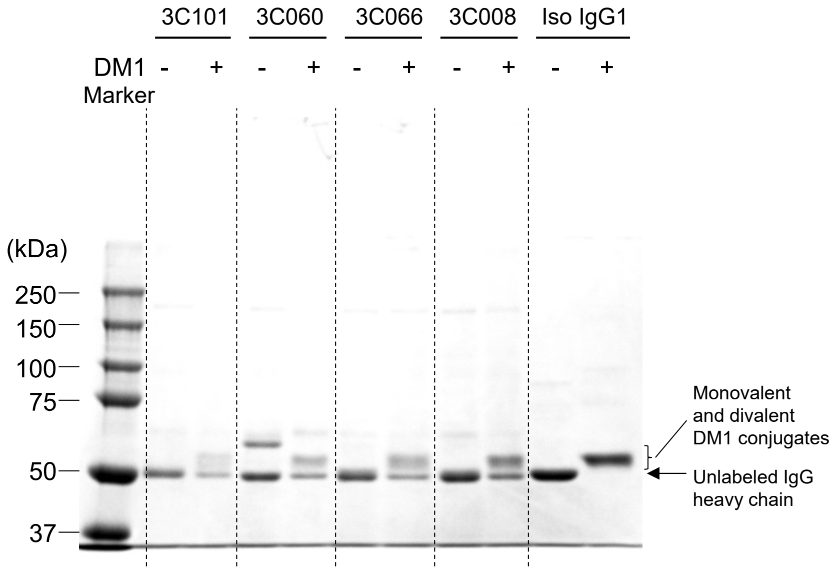

Supplementary Figure 11. Uncropped images of SDS-PAGE.
Uncropped images of SDS-PAGE analysis are presented. The figure used in Supplementary Fig. 6.

Individual A

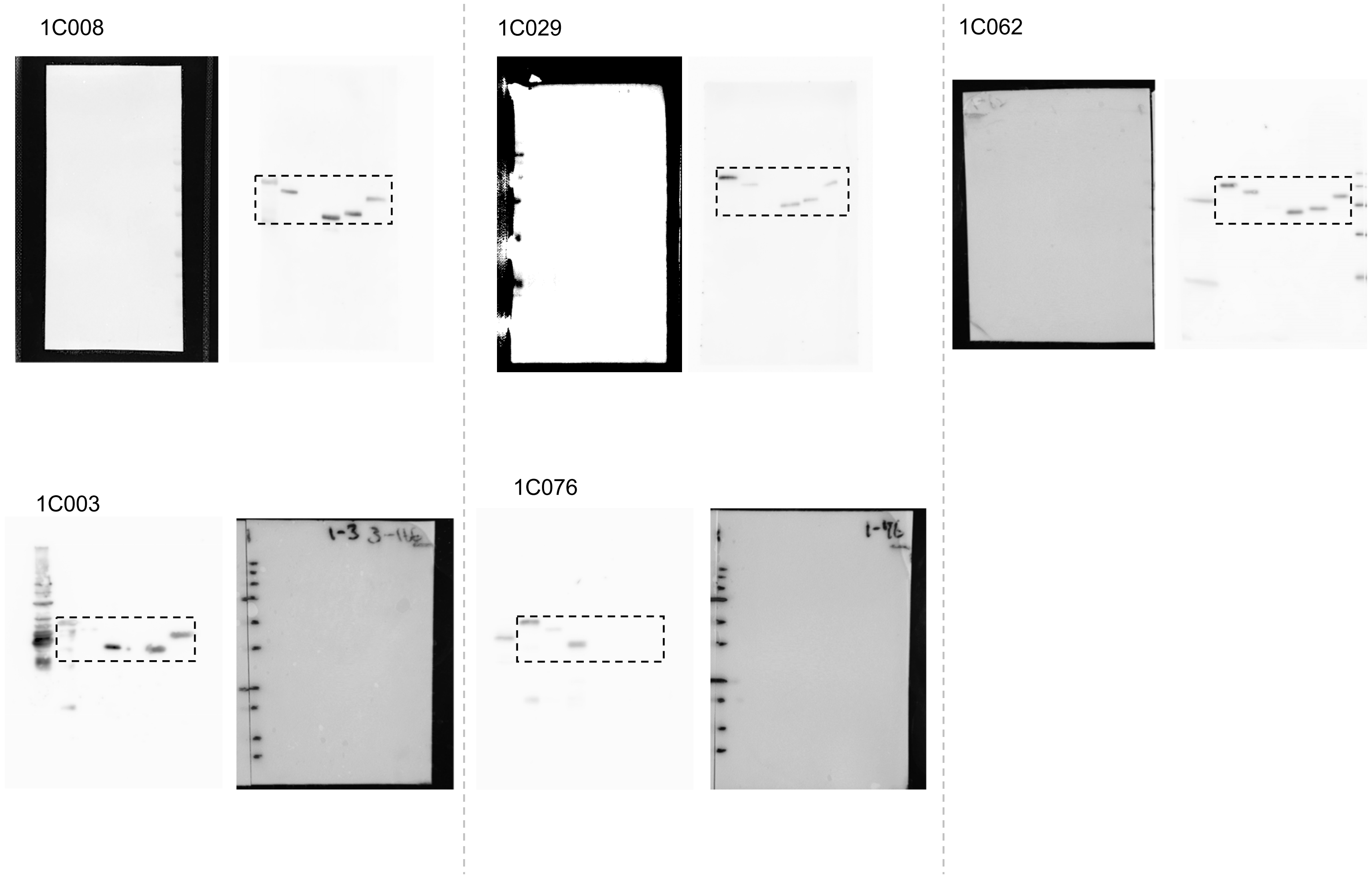

Individual B

3C008

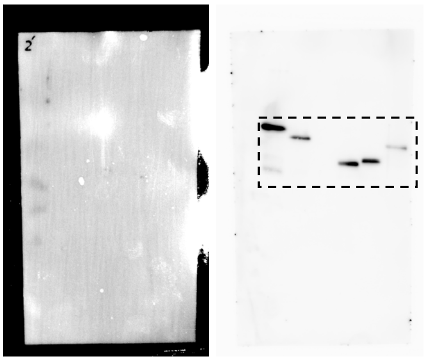

3C024

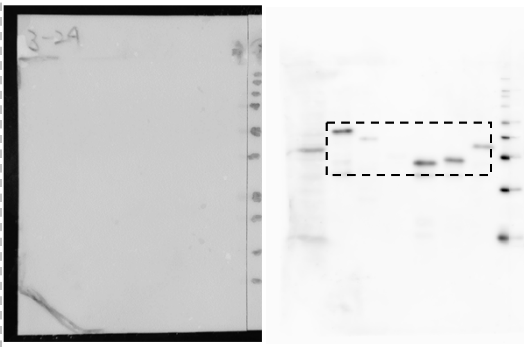

3C028a

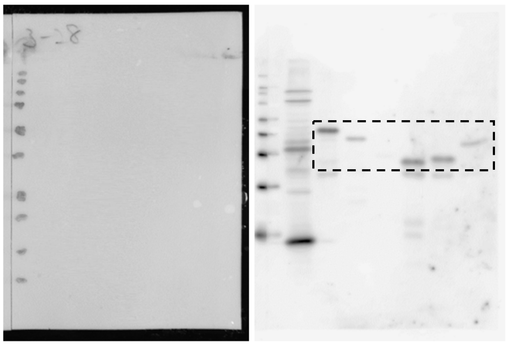

3C035

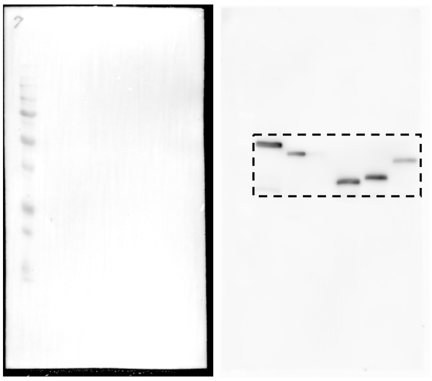

3C044

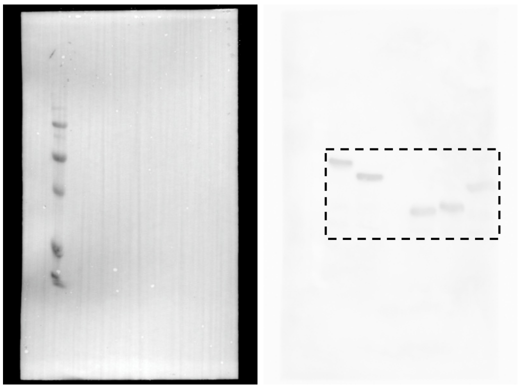

3C049

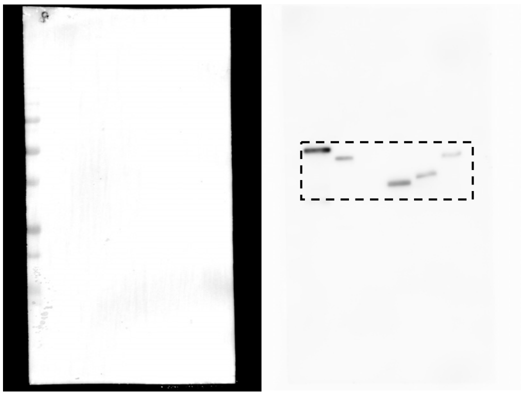

3C052

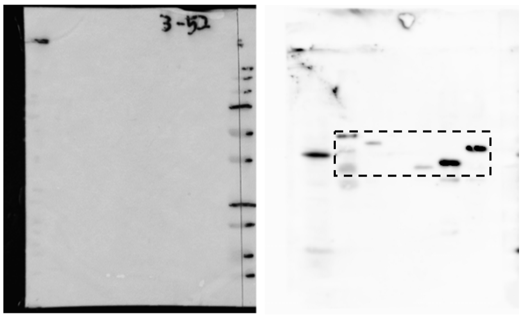

3C058

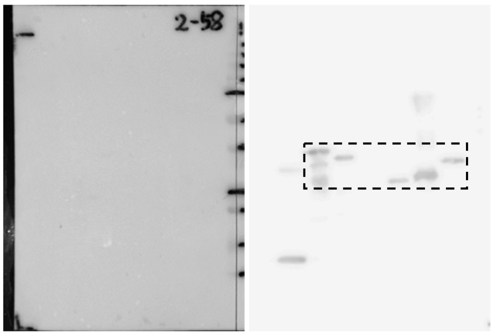

3C060

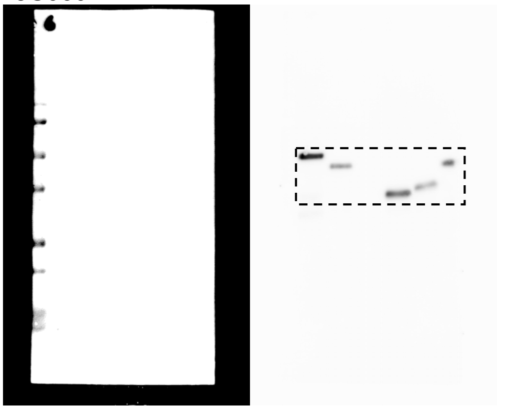

3C068

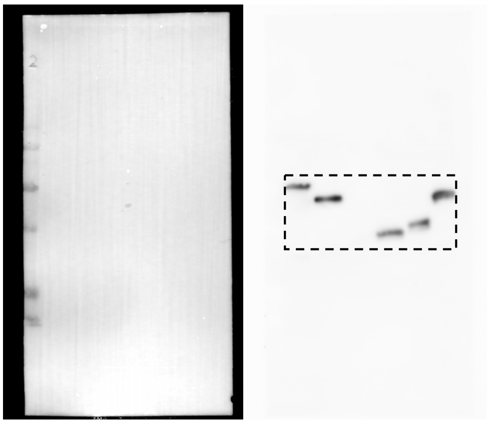

3C076

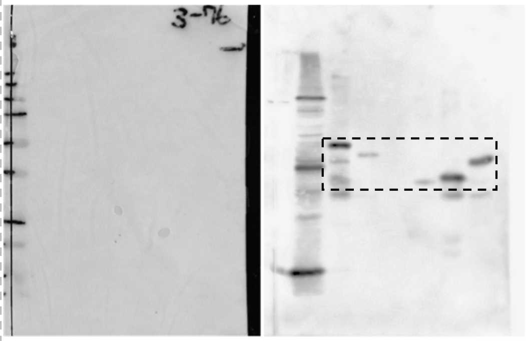

3C101

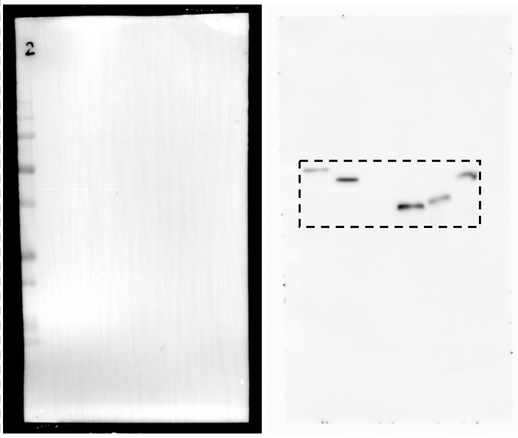

(continued)

Individual B

3C109

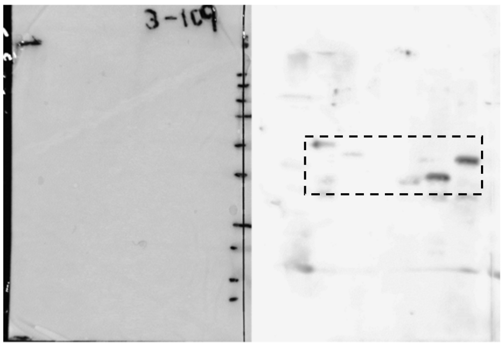

3C124

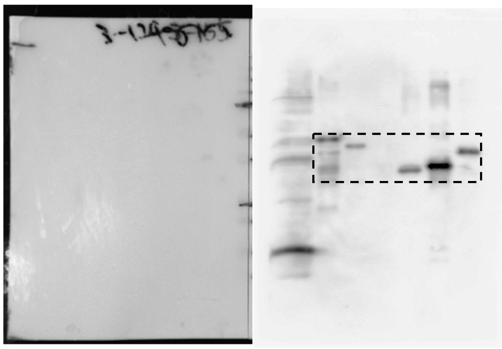

3C138

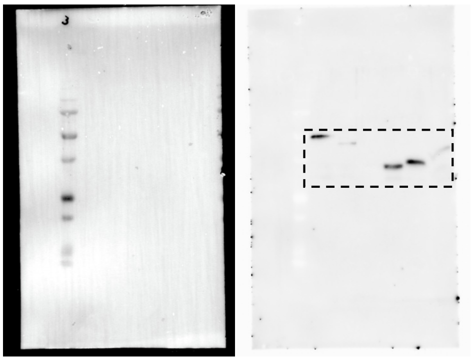

3C145

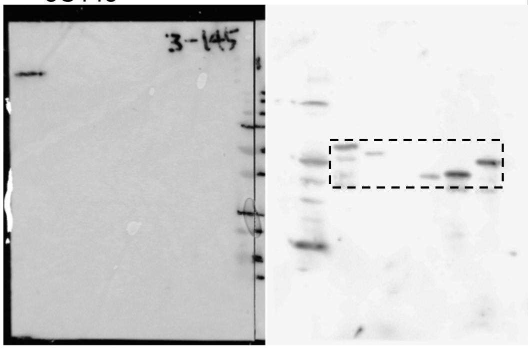

3C146

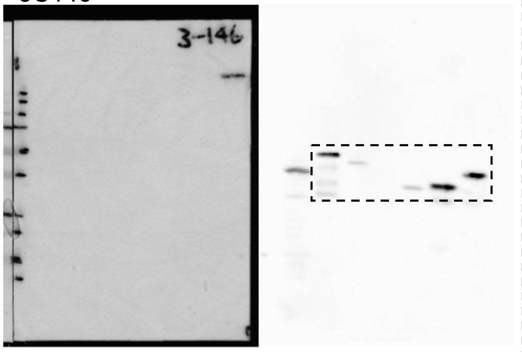

3C155

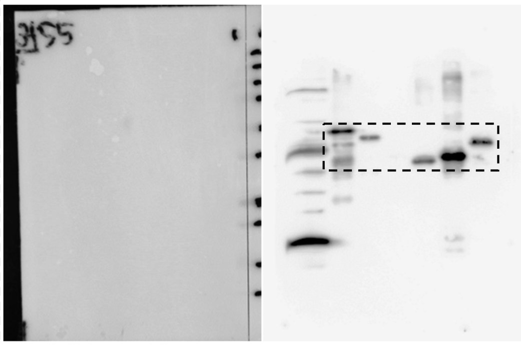

3C159

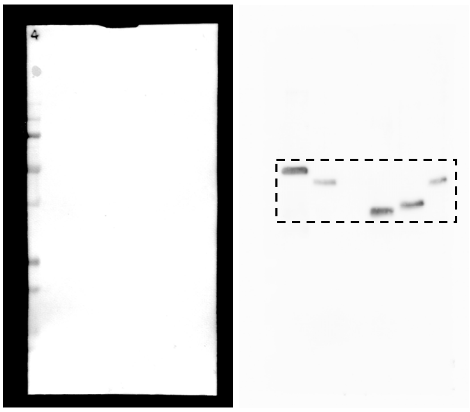

3C164

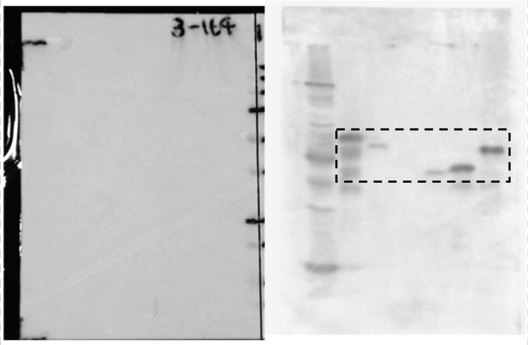

3C166

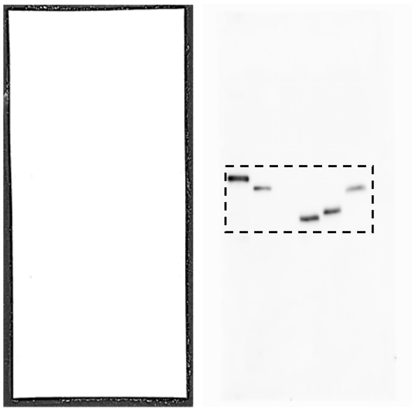

3C169

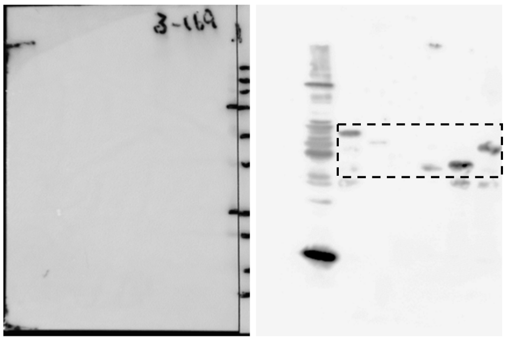

3C173

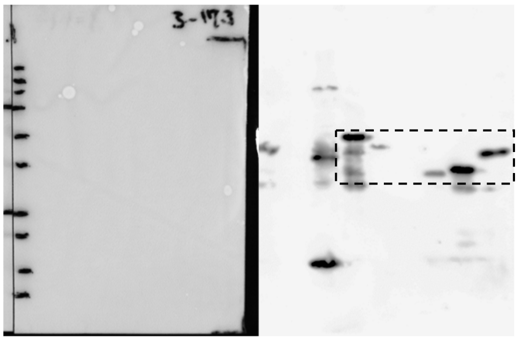

3C194

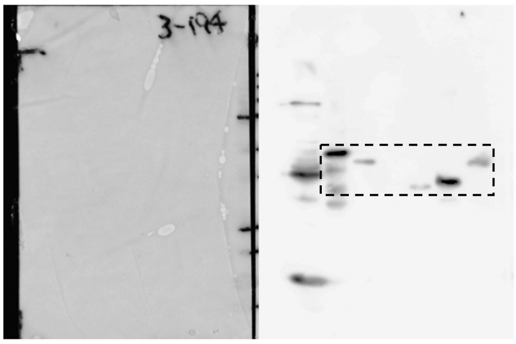

Individual B

3C202

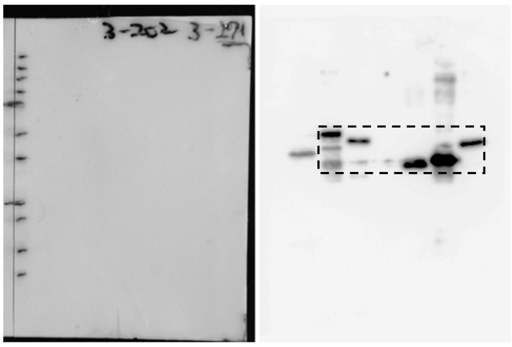

3C213

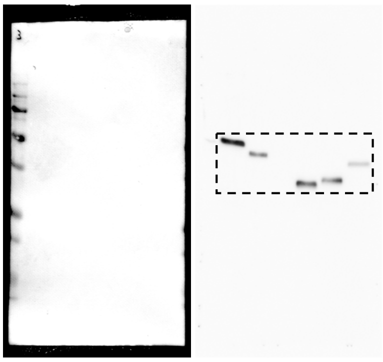

3C214

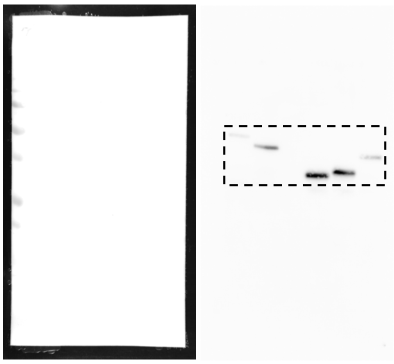

3C227

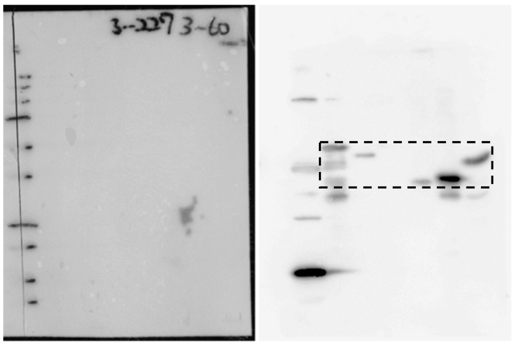

3C232

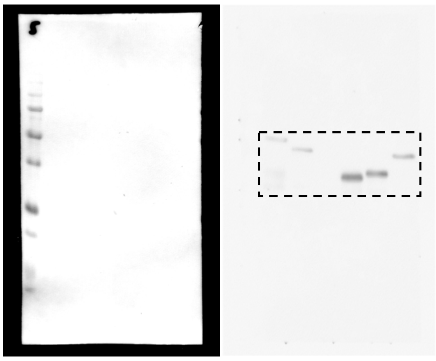

3C248

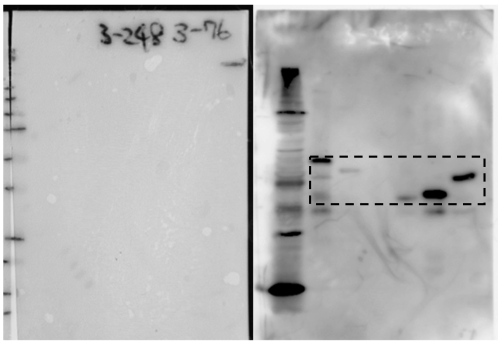

3C250

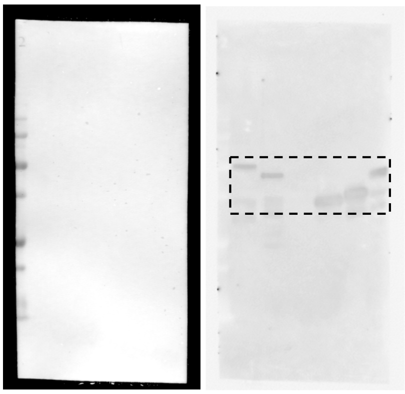

3C256

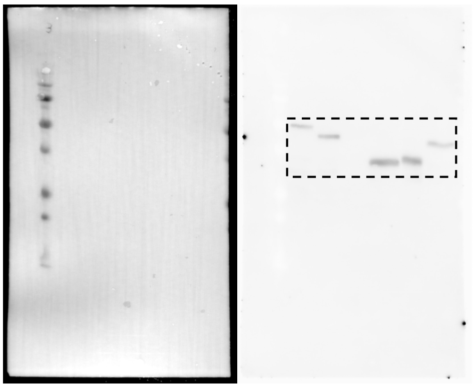

3C260

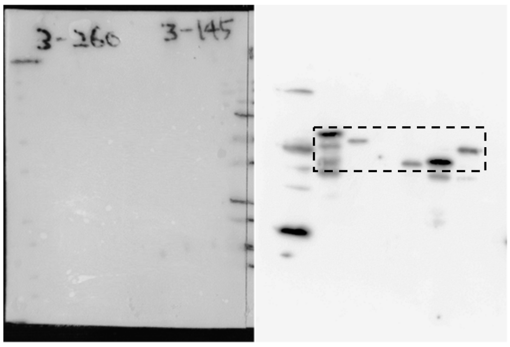

3C266

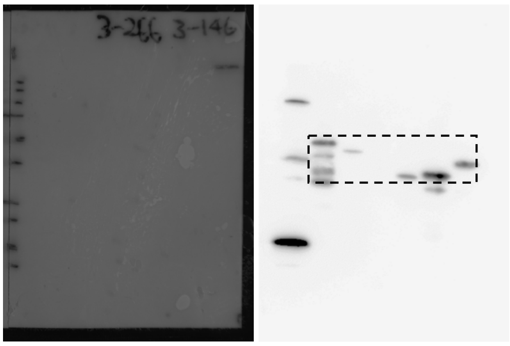

3C286

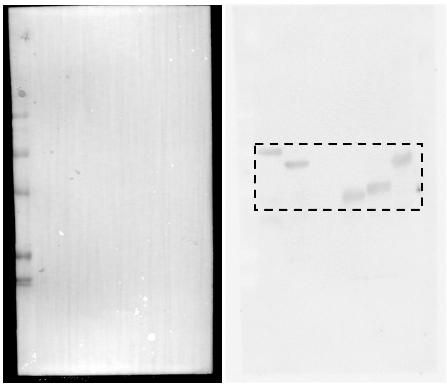

3A287

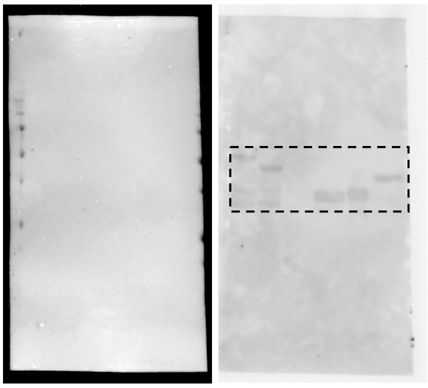

Individual B

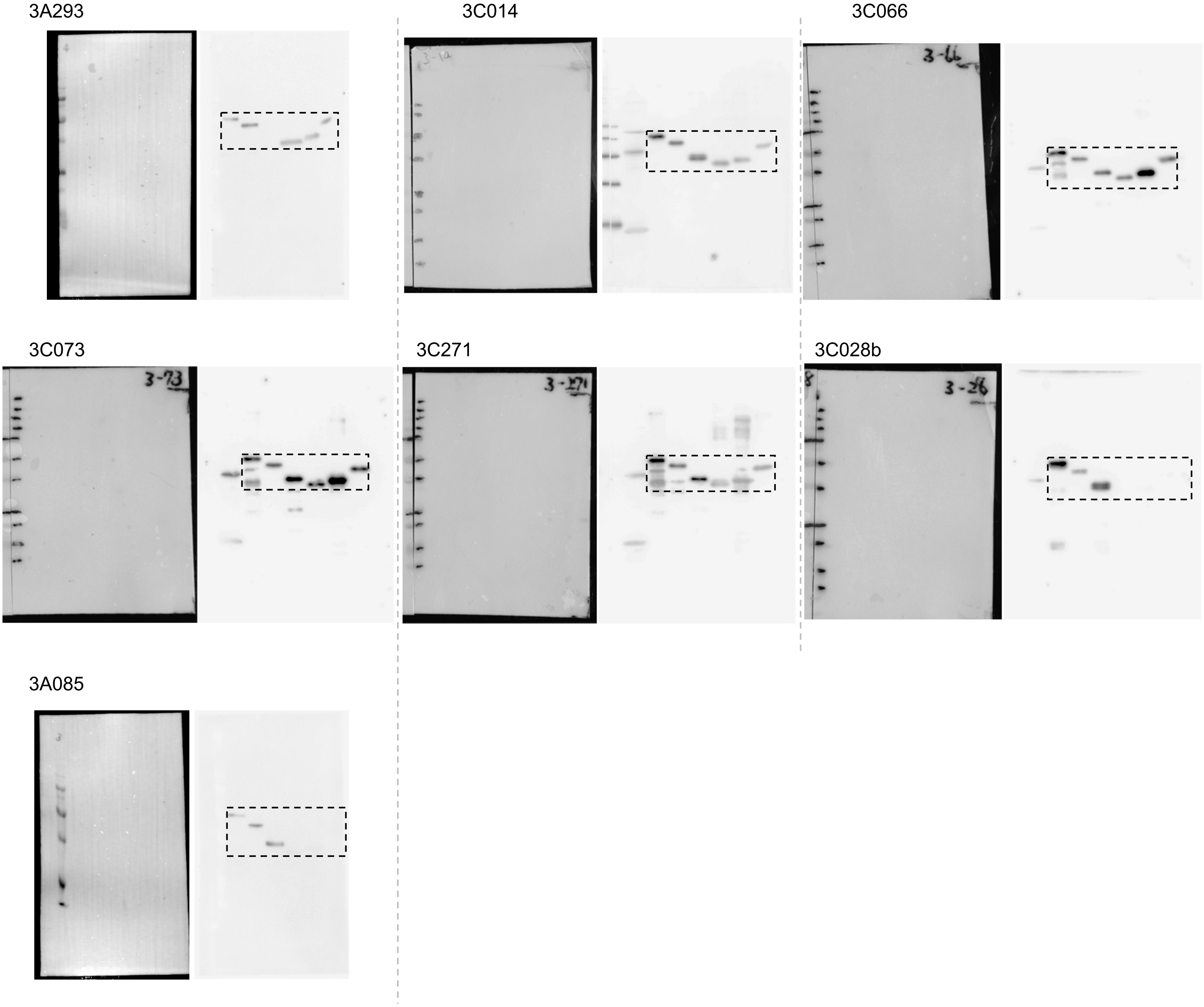

Supplementary Figure 12. Uncropped images of western blotting.
Uncropped images of all WB analyses are presented. In each clone shown in the upper left, the left image shows the membrane in visible light, and the right image shows the chemiluminescence detected. The dotted square indicates the figure used in Supplementary Fig. 10. The case where the signal is seen to the left of dotted square is analyzed for HCT116 whole extract. If a ladder is seen on either side of the image, it is a molecular weight marker.

Supplementary Table 1. Primer design

Target sequence	Forward primer	Reverse primer
**Cloning of a partial region of EpCAM**		
EpEX	5′-AAAGATATCGGATCCTCAGGAAGAATGTGTCTGTGA-3′	5′-ATAAAGCTTTTTTAGACCCTGCATTGAGAATTC-3′
EpRE	5′-AAAGGATCCAAGAGCAAAACCTGAAGG-3'	5′-ATAAAGCTTTTTTAGACCCTGCATTGAGAATTC-3′
EpN62	5′-AAAGGATCCGCTGGCTGCCAAATGTTTG-3'	5′-ATAAAGCTTTTTTAGACCCTGCATTGAGAATTC-3′
EpC162	5′-AAAGATATCGGATCCTCAGGAAGAATGTGTCTGTGA-3′	5′-ATAAAGCTTCAAACTTTTACTATCATAAGGTTTTTCTC-3'
EpC184	5′-AAAGATATCGGATCCTCAGGAAGAATGTGTCTGTGA-3′	5'-ATAAAGCTTAATACTCGTGATAAATTTTGGATCCAG-3'
EpC218	5′-AAAGATATCGGATCCTCAGGAAGAATGTGTCTGTGA-3′	5'-ATAAAGCTTTTTTTCAAAATAATAAGCCACATCAGC-3'
**Cloning of Ab variable region (5'-RACE)**		
H chain		5'-CAGGGGTCCGGGAGATCATGAGGGTG-3'
L chain		5'-GTGCTGTCCTTGCTGTCCTGCT-3'

Underlined sequences indicate the restriction enzyme cutting sites.
